# MicroRNAs as Biomarkers and Therapeutic Targets in Treatment-Resistant Depression: Unveiling Diagnostic and Treatment Pathways

**DOI:** 10.2174/0127724328381443250825092900

**Published:** 2025-09-01

**Authors:** Alaa A. A. Aljabali, Almuthanna K. Alkaraki, Omar Gammoh, Esam Qnais, Abdelrahim Alqudah, Walhan Alshaer, Vijay Mishra, Yachana Mishra, Mohamed El-Tanani

**Affiliations:** 1 Faculty of Pharmacy, Department of Pharmaceutics and Pharmaceutical Technology, Yarmouk University, Irbid 21163, Jordan;; 2 Department of Biological Sciences, Faculty of Science, Yarmouk University, Irbid 21163, Jordan;; 3 Department of Clinical Pharmacy and Pharmacy Practice, Faculty of Pharmacy, Yarmouk University, PO BOX 566, Irbid, 21163, Jordan;; 4 Department of Biology and Biotechnology, Faculty of Science, The Hashemite University, Zarqa, Jordan;; 5 Department of Clinical Pharmacy and Pharmacy Practice, Faculty of Pharmaceutical Sciences, The Hashemite University, Zarqa 13133, Jordan;; 6 Cell Therapy Center, University of Jordan, Amman, Jordan;; 7 School of Pharmaceutical Sciences, Lovely Professional University, Phagwara (Punjab) 144411, India;; 8 School of Bioengineering and Biosciences, Lovely Professional University, Phagwara (Punjab) 144411, India;; 9 College of Pharmacy, Ras Al Khaimah Medical and Health Sciences University, Ras Al Khaimah, UAE

**Keywords:** Treatment-resistant depression (TRD), MicroRNAs (miRNAs), biomarkers, miRNA-based therapeutics, neuroinflammation, precision psychiatry, antidepressant resistance

## Abstract

**Introduction:**

Treatment-Resistant Depression (TRD) is a complex clinical condition characterized by inadequate response to conventional antidepressant treatments. There is growing evidence that microRNAs (miRNAs) play a role in the underlying pathophysiology of TRD and may offer new avenues for diagnostics and therapy.

**Methods:**

A structured literature review of peer-reviewed publications indexed in PubMed, Scopus, and Web of Science was conducted. The search strategy included combinations of keywords such as “treatment-resistant depression,” “microRNAs,” “biomarkers,” and “miRNA-based interventions.” Articles were selected based on relevance to miRNA expression patterns in TRD, therapeutic modulation, and their clinical potential.

**Results:**

Dysregulation of several miRNAs including miR-135a, miR-34a, and miR-155 was consistently observed in patients with TRD. These miRNAs were linked to impaired synaptic plasticity and persistent neuroinflammation. Therapeutic approaches using miRNA mimics or inhibitors showed potential in restoring neurobiological balance and enhancing response to traditional antidepressants. However, delivery system limitations and blood-brain barrier penetration remain significant challenges.

**Discussion:**

miRNAs appear to play a dual role in TRD, serving both as biomarkers for diagnosis and as targets for novel therapies. Integrating miRNA profiling into clinical workflows could enhance diagnostic precision and guide individualized treatment strategies. Translational barriers, such as delivery specificity and standardization of detection protocols, must be addressed before the widespread clinical application of this technology.

**Conclusion:**

This review highlights miRNAs as promising diagnostic and therapeutic tools in TRD. Continued advancements in delivery systems and validation of biomarker panels may pave the way for their clinical implementation in personalized psychiatry.

## 1 INTRODUCTION

### 1.1 Overview of Major Depressive Disorder (MDD)

Major Depressive Disorder (MDD) is a leading global psychiatric condition characterized by persistent low mood, anhedonia, and functional impairment [1]. According to the World Health Organization, it affects over 264 million individuals worldwide and remains a principal cause of disability [2, 3]. Among patients diagnosed with MDD, approximately 20–30% develop Treatment-Resistant Depression (TRD), a severe form defined by a lack of response to at least two antidepressant regimens administered at an adequate dose and duration [1, 4, 5]. TRD is associated with greater psychiatric comorbidity, suicidality, and significant socioeconomic burden. Prevalence estimates vary across regions, with figures ranging from 4.7% in Oceania to 31.9% in Africa [6-8].

MDD is a psychiatric condition characterized by persistent sadness and feelings of hopelessness as well as a lack of interest or pleasure in previously enjoyed activities [4]. Current estimates of MDD prevalence show significant variability across populations and geographical regions, ranging from 4.7% in Oceania to 31.9% in Africa. This heterogeneity suggests a significant impact of various socioeconomic, cultural, and environmental factors on the emergence of the disorder [9-11].

Emerging research suggests that microRNAs, which are small non-coding RNAs, have the potential to act as post-transcriptional regulators of gene expression and may provide novel insights into the understanding and treatment of TRD [12-17]. These molecules can influence diverse biological pathways, including those associated with inflammation, neurogenesis, and stress response mechanisms, all of which have been implicated in TRD pathology [16]. Their presence in the peripheral blood and CSF makes them a promising avenue for noninvasive biomarkers, while their regulatory capabilities make them potential therapeutic targets [17, 18]. Biomarkers of neuroinflammation are important for understanding the underlying mechanisms of TRD. These include cytokines, acute-phase proteins, neuroimmune enzymes, oxidative stress indicators, glial activation markers, immune cell phenotypes, and hormone-related metabolites, as shown in Table **1**. Together, they reflect the complex interplay between immune dysregulation, metabolic imbalances, and neuroinflammatory processes in TRD.

The heterogeneous nature of this disorder complicates its diagnosis and treatment. MDD can manifest as polymorphic variants, owing to the predominance of affective disturbances in cognitive disorders and somatic complaints [19]. This variability is further complicated by the broad range of individual responses to treatment, which may also range from pharmacotherapy to psychotherapy and lifestyle modifications [20]. Common interventions include antidepressants, often in the form of SSRIs, and CBT. However, there is considerable interindividual variability in treatment response, with a significant subset of patients experiencing inadequate symptom relief [21, 22].

A particularly formidable subset of the MDD continuum is Treatment-Resistant Depression (TRD), defined as the failure to respond to at least two antidepressant treatments of adequate dose and duration [23]. Approximately 30% of patients with MDD meet the definition of TRD, which poses a significant public health burden. Patients who develop TRD tend to be more symptomatic, have higher comorbidity and illness levels, and have greater functional impairment than treatment responders [7]. TRD complicates clinical management and often necessitates higher-intensity treatment strategies, including medication combinations, augmentation strategies with typical antipsychotics or mood stabilizers, and novel interventions, such as TMS or ECT [24]. Emerging evidence suggests an implicated neuroinflammatory pathway in the pathophysiology of TRD, characterized by frequently observed elevated levels of various cytokines, including IL-6 and TNF-α, in patients who do not respond to antidepressant therapies. The broader implications of TRD affect not only individual patients but also place a sustained burden on healthcare systems and clinical infrastructure. The next section presents a SWOT analysis, outlining the strengths, weaknesses, opportunities, and threats relevant to current diagnostic and therapeutic practices in TRD research. This is associated with increased healthcare utilization and elevated suicide risk. Therefore, understanding the underlying mechanisms contributing to TRD is essential for the development of effective treatment strategies [25].

In recent years, miRNAs have been recognized as crucial regulatory molecules in the field of psychiatry, particularly through their roles in the neurobiological processes underlying MDD and TRD [7, 16]. miRNAs are small (approximately 19-24 nucleotides long) non-coding RNA molecules that regulate gene expression at the post-transcriptional level by interacting with complementary sequences on target messenger RNAs [26, 27]. The ability of miRNAs to modulate various biological processes, such as neural development and synaptic plasticity, and their responses to stress, is well established. Their dysregulation is increasingly linked to pathophysiological mechanisms underlying depressive disorders [28]. There is evidence that miRNAs play a role in neuroplasticity, the brain's capacity to change structurally and functionally in response to experience, through the regulation of gene expression related to synaptic transmission and neurogenesis [28, 29].

Dysregulation of miRNA expression has also been linked to inflammatory processes, which are frequently observed in MDD patients. For example, pro-inflammatory cytokines may alter the expression profile of miRNAs, further promoting depressive symptoms [29, 30]. The interplay between stress and miRNA regulation is an important research topic in this field. Chronic stress affects both miRNA expression and neurobiological pathways involved in mood regulation. This indicates that alterations in miRNA levels could be potential biomarkers for identifying individuals at risk of developing depression or those who may have inadequate responses to conventional treatments [31-33]. Fig. (**1**) presents a miRNA–disease interaction network identifying miRNAs linked to major depressive subtypes, including postpartum and post-stroke depression. Specific to the constructed network suggests that miRNAs act as central regulatory modulators in controlling pathways specific to certain diseases.

Therefore, targeting miRNAs may provide significant therapeutic benefits. Restoration of normal miRNA function or inhibition of dysregulated miRNAs may improve neuroplasticity and lead to better treatment outcomes in patients with MDD and TRD [34, 35]. However, understanding the specific delivery mechanisms of miRNA-based therapies remains challenging. In summary, MDD is a serious health disorder owing to its heterogeneity and mixed presentation, with highly variable treatment needs and responses that complicate clinical management [34, 36]. This landscape is further complicated by situations such as Treatment-Resistant Depression, which require innovative management approaches. The emerging role of miRNAs offers important molecular insights into understanding the molecular underpinnings of depression. It may point to novel therapeutic strategies aimed at improving outcomes for individuals affected by the condition [37, 38]. Further research on the interplay between miRNAs, neuroplasticity, inflammation, and stress response is needed to improve our understanding and treatment of debilitating disorders [39]. Fig. (**2**) illustrates the signaling pathway of BDNF, a major regulator of neuroplasticity whose dysregulation has been related to disrupted neuronal connectivity and reduced synaptic function, salient features of treatment-resistant depression.

Synaptic plasticity, the brain's ability to change neural connections based on experience, is a mechanism considered critical for learning and memory, and it appears to be profoundly affected in TRD [39, 40]. Specific miRNAs, including miR-34a and miR-124, have been implicated in synaptic function and neurogenesis. Synaptic plasticity, the brain's capability to change neural connections based on experience, is a mechanism considered critical in the performance of learning and memory, and it seems profoundly affected in TRD [37, 38]. Specific miRNAs, including miR-34a and miR-124, have been implicated in synaptic function and neurogenesis, of which miR-34a was clearly demonstrated to be upregulated in TRD. miR-34a exerts its effects through genes that regulate synaptic pruning and dendritic spine morphology, which are critical for synaptic connectivity and stability [39]. Furthermore, miR-124, which is generally downregulated in TRD, controls the levels of Brain-Derived Neurotrophic Factor (BDNF) and other neurotrophic factors necessary for neuronal survival and plasticity [40]. Therefore, alterations in miR-124 expression could underlie the impaired neuroplastic response observed in treatment-resistant depression, in which patients fail to respond adaptively to antidepressants [41]. Another miRNA of interest in TRD is miR-132, which modulates the CREB-BDNF signaling axis. Reduced miR-132 expression has been associated with impaired dendritic arborization and disrupted synaptic remodeling, particularly in prefrontal and hippocampal regions of patients with antidepressant resistance [42]. Its dysregulation contributes to abnormal neuroplasticity under chronic stress exposure, in the context of neuroinflammation. In this context, miR-146a functions as a post-transcriptional inhibitor of the NF-κB signaling pathway by targeting the TRAF6 and IRAK1 proteins. Its downregulation in TRD models has been shown to perpetuate microglial activation and sustained proinflammatory cytokine production, which may interfere with antidepressant efficacy [43]. Additionally, miR-124 has been implicated in endocrine regulation, particularly by targeting the NR3C1 gene, which encodes the glucocorticoid receptor. Overexpression of miR-124 has been linked to reduced glucocorticoid receptor sensitivity and impaired Hypothalamic–Pituitary–Adrenal (HPA) axis feedback, potentially contributing to cortisol dysregulation observed in TRD [44]. miR-34a exerts its effects through genes that regulate synaptic pruning and dendritic spine morphology, which are critical for synaptic connectivity and stability [41]. Furthermore, miR-124, which is generally downregulated in TRD, controls the levels of Brain-Derived Neurotrophic Factor (BDNF) and other neurotrophic factors necessary for neuronal survival and plasticity [42]. Therefore, alterations in miR-124 expression could underlie the impaired neuroplastic response observed in treatment-resistant depression, in which patients fail to respond adaptively to antidepressants [43]. Another miRNA of interest in TRD is miR-132, which modulates the CREB-BDNF signaling axis. Reduced miR-132 expression has been associated with impaired dendritic arborization and disrupted synaptic remodeling, particularly in prefrontal and hippocampal regions of patients with antidepressant resistance [44, 45].

To consolidate the mechanistic roles of miRNAs implicated in TRD, we have summarized key regulatory targets and associated biological processes in Table **2**. The included miRNAs span neuroinflammatory, neuroplastic, and endocrine pathways known to be disrupted in resistant depression phenotypes. Fig. (**3**) presents a scatterplot of the expression levels of genes implicated in treatment-resistant depression. Key genes include BDNF, IL6, and TNF, which are differentially expressed as a result of disruptions in neuroinflammatory and neuroplasticity pathways.

This review appraised the mechanistic roles of miRNAs in TRD and discussed their implications for diagnostics, biomarkers, and therapeutic interventions from the broader perspective of treatment-resistant psychiatric disorders. TRD continues to present a significant clinical challenge, necessitating a deeper understanding of its biological complexity. The SWOT analysis in Table **2** summarizes the strengths, weaknesses, opportunities, and threats related to the current research and therapeutic strategies for TRD. These clinical challenges necessitate innovative molecular approaches in studies, such as those on miRNAs, which hold promise for elucidating and addressing the multifactorial pathophysiology of TRD.

### 1.2 Methodology

This review followed a narrative synthesis design, structured to comprehensively explore the role of microRNAs (miRNAs) in Treatment-Resistant Depression (TRD). Relevant literature was identified through systematic searches across major scientific databases, including PubMed, Scopus, Web of Science, and Google Scholar. The search covered publications from 2000 to 2024, using combinations of keywords such as "microRNA", "miRNA", "treatment-resistant depression", "TRD", "biomarkers", "neuroinflammation", and "synaptic plasticity".

Articles were included based on their focus on human studies, preclinical models, or translational applications specifically related to the roles of miRNA in TRD. Reviews, original research, and meta-analyses were prioritized. Studies discussing miRNA mechanisms in related psychiatric or neuroinflammatory contexts were also considered when relevant to the pathophysiology of TRD or therapeutic insights.

Duplicate records, non-English publications, non-peer-reviewed articles, and those lacking sufficient methodological detail were excluded. The included literature was then categorized by thematic relevance into miRNA mechanisms, targets, biomarker potential, and therapeutic implications. Emphasis was placed on recent high-impact studies, particularly those published in the last five years.

## 2 MECHANISTIC ROLE OF MIRNAS IN DEPRESSION AND TRD

### 2.1 miRNAs in Neuroinflammation

miRNAs are a class of small non-coding RNAs that post-transcriptionally regulate gene expression, primarily by binding to messenger RNAs (mRNAs) and modulating their stability or translation. In the context of neuroinflammation, miRNAs have emerged as key regulators, with specific examples, such as miR-146a, demonstrating the ability to modulate immune responses by influencing cytokine production and other pathways critical to immune regulation [47]. TRD is strongly associated with persistent neuroinflammation and dysregulated serotonin signaling, both of which contribute to its pathophysiology. Fig. (**1**) illustrates the two critical mechanisms through which miRNAs regulate this process. Fig. (**4A**) shows the function of miR-146a in modulating TLR signaling. It inhibits NF-κB signaling by targeting TRAF6 and IRAK1, resulting in the inactivation of NF-κB and a reduction in the production of pro-inflammatory cytokines, including IL-6 and TNF-α. The detailed mechanism forms the basis of how miR-146a contributes to reducing neuroinflammation, a feature of TRD. In addition to miR-146a, miR-155-3p has been identified as a key modulator of neuroinflammation and neuroplasticity pathways in patients with TRD. Fig. (**4B**) illustrates that the target genes of miR-155-3p, including TP53, PTEN, and SIRT1, are implicated in disrupting cellular homeostasis and perpetuating depressive symptoms.

Collectively, the regulatory activity of miR-155-3p through these targets underlines its core role in disrupting cellular and molecular homeostasis in TRD. Overexpression of miR-155-3p has been linked to the amplification of neuroinflammatory pathways, a reduction in neuroplasticity, and decreased neuronal survival. Such disruptions directly affect the persistence of depressive symptoms and resistance to conventional antidepressant therapies. MiR-155-3p is increasingly recognized as both a biomarker of disease severity and a therapeutic target in TRD. Due to its involvement in key pathogenic processes, miR-155-3p holds dual relevance for diagnosis and treatment. Modulating its expression may interrupt downstream dysregulation and improve therapeutic outcomes in TRD. Fig. (**4**) provides schematic illustrations of the two critical mechanisms by which miRNAs regulate processes central to TRD pathology. Panel A illustrates the role of miR-146a in regulating inflammatory pathways. MiR-146a represses NF-κB activation by targeting the adapters TRAF6 and IRAK1, thus reducing the expression of pro-inflammatory cytokines, such as IL-6 and TNF-α. This mechanism reduces the neuroinflammatory response commonly associated with TRD, thereby linking the dysregulation of miR-146a to the pathogenesis. In TRD, miRNAs regulate key pathways associated with neuroplasticity, inflammation, and cellular stress responses by targeting specific genes and proteins.

Fig. (**4B**) illustrates the involvement of miRNAs in the serotonin signaling pathway. For example, miR-135a targets the serotonin transporter gene, SLC6A4, which regulates serotonin reuptake and enhances the efficacy of SSRIs. Serotonin is catabolized by monoamine oxidase A (MAOA), which is also targeted by miRNAs, further highlighting their role in serotonin metabolism. These two parallel regulatory mechanisms provide not only an explanation for serotonergic dysfunction in TRD but also support the use of miRNAs as potential therapeutic targets in the development of more effective antidepressants.

To further investigate the role of miRNAs in TRD, their target genes, proteins, and associated pathways were examined using KEGG and miRNet analyses. Some of the key miRNAs include miR-1202 and miR-124, implicated in neuroplasticity; miR-146a in inflammation; and miR-21 in apoptosis. For example, miR-146a is associated with enhanced inflammatory response, whereas miR-124 is associated with impaired neurogenesis. MiRNAs, including miR-146a and miR-155-3p, target the NF-κB signaling pathway and neuroplasticity, among other critical pathways, making them key therapeutic targets for TRD, as shown in Table **3**. Table (**3**) provides a summary of miRNA involvement in TRD, including their putative gene and protein targets and related biological functions. This table represents the regulatory functions that specific miRNAs play in fundamental pathways, such as neuroplasticity, inflammation, and apoptosis-all these processes represent core pathology in TRD. miR-1202 and miR-124 contribute to synaptic remodeling and neuroplasticity, which are critical processes responsible for healthy neuronal connectivity. MiR-146a is most notable for its anti-inflammatory role, particularly in the inhibition of pro-inflammatory cytokine production, targeting key signaling adapters such as TRAF6 and IRAK1. Additionally, other miRNAs, such as miR-16 and miR-21, have been shown to influence apoptotic pathways and modulate cellular survival in response to stress. Taken together, these findings consolidate the evidence that miRNAs regulate molecular and cellular mechanisms central to TRD and may support the development of adjunctive therapies aimed at restoring disrupted pathways through miRNA targeting.

Recent research has highlighted that miR-155-3p is a significant modulator of pathways implicated in the pathophysiology of TRD. miR-155-3p targets a diverse set of genes, as illustrated in Fig. (**5**), and is implicated in cellular stress responses, apoptosis, and neuroinflammation, which are key molecular processes associated with TRD. These mechanisms collectively contribute to the underlying biology of treatment resistance, which is a key target of miR-15. One of the key targets of miR-155-3p is TP53, a tumor suppressor gene that plays a crucial role in cellular stress response and apoptosis. Dysregulation of TP53 in TRD enhances neuronal loss, further amplifying depressive symptoms. The disruption of this pathway contributes to the progressive nature of TRD.

Another target of interest is PTEN, a key gene in the PI3K/AKT signaling pathway that is crucial for regulating neuroplasticity and cell survival. Dysregulation of PTEN by miR-155-3p may impair synaptic plasticity, further facilitating cognitive-emotional dysfunction in TRD. Moreover, SIRT1, which is involved in neuronal protection by enhancing synaptic plasticity and controlling oxidative stress, is targeted by miR-155-3p. These data suggest that miRNAs play a crucial role in protecting against neuronal damage and contribute to maintaining resilience. ERBB2 plays a role in neuronal survival and repair. The modulation of ERBB2 by miR-155-3p disrupts neuroprotective processes and contributes to the pathophysiology of TRD.

Neuroinflammation plays a crucial role in the pathogenesis of Treatment-Resistant Depression (TRD), and microRNAs (miRNAs) are critical regulators of this process. Fig. (**6A**) demonstrates the enrichment of KEGG pathways related to cytokine–cytokine receptor interaction and Toll-like receptor signaling, highlighting the involvement of miR-146a and miR-124 in modulating innate immune responses. Specifically, Fig. (**6B**) illustrates the suppression of NF-κB signaling through targeting of TRAF6 and IRAK1, both of which are upstream regulators of pro-inflammatory cytokines. The MAPK signaling pathway and JAK-STAT signaling, as shown in Fig. (**6C**), are also modulated by miR-146a and contribute to the downstream expression of inflammatory genes. Finally, Fig. (**6D**) shows the gene count and statistical significance of each enriched pathway, further supporting the regulatory roles of these miRNAs in pathways relevant to neuroinflammatory responses in TRD. Together, these analyses indicate that miR-146a decreases pro-inflammatory cytokines and reduces neuroinflammation by targeting TRAF6 and IRAK1, which has been linked to an improvement in behavioral symptoms associated with TRD.

- miRNAs influence neuroplasticity: MiRNAs that affect neurogenesis, synaptic plasticity, and neuronal survival are crucial in the brain's adaptive responses to chronic stress and depression. Notable examples include miR-124, which impacts neuroplasticity by targeting proteins such as BDNF and synaptic proteins [48-50].
- miRNAs modulate the HPA axis in TRD: outline how miRNAs influence the hypothalamic-pituitary-adrenal (HPA) axis. This includes miRNAs such as miR-34c, which modulates stress hormone receptors [51-53]. MiRNAs have dual roles as biomarkers for the diagnosis and prediction of TRD. The interaction map demonstrates complex regulatory relationships among miRNA and genes, with miR-135a and miR-146a emerging as 'hubs.’ High levels of miR-146a correlate with inflammatory markers and serve a diagnostic role, whereas high levels of miR-135a predict treatment with SSRIs due to modulation of serotonin transporter expression. This added functionality reinforces the importance of miRNAs in advancing precise psychiatric approaches for TRD treatment.

### 2.2 The Mechanistic Role of miRNAs in Depression and Treatment-Resistant Depression (TRD)

Neuroinflammation is increasingly recognized as a crucial pathophysiological feature of TRD, and miRNAs are increasingly acknowledged as critical regulators within this inflammatory context. For instance, other miRNAs reported in the literature to mediate inflammatory responses include miR-146a and miR-155, which target components of the NF-κB pathway involved in regulating cytokine production and the immune response [51, 54]. Specifically, miR-146a negatively regulates IRAK1 and TRAF6, thereby controlling excessive inflammatory signals that correlate with depression severity, as summarized in Table **4**. In contrast, the overexpression of miR-155 promotes pro-inflammatory signaling by enhancing TNF-α expression, a cytokine implicated in depressive symptoms [55]. TRD, where inflammation is more often chronic, dysregulation of these miRNAs could exacerbate the inflammatory state to sustain or intensify depressive symptoms [56-58].

MiRNAs are small noncoding RNA molecules that regulate gene expression. They have been implicated in neuroinflammation, neuroplasticity, and stress response and have been suggested in the context of MDD and TRD [59, 60]. Given their role as regulators, miRNAs are potential biomarkers and therapeutic targets in psychiatric disorders, with specific miRNAs such as miR-146a, miR-124, and miR-34c emerging as key players in MDD and TRD. Subsequent sections examine the mechanistic role of these miRNAs and describe how they affect the pathways integral to the pathophysiology of depression [61-64]. To explain how specific miRNAs regulate neuroinflammation, researchers have concurrently identified target molecules that could be harnessed for therapeutic purposes, particularly in patients with TRD who are unresponsive to standard treatments [29].

### 2.3 Role of miR-146a in Inflammatory Pathways

Neuroinflammation is increasingly recognized as a critical factor in the pathophysiology of depression, exacerbating symptom severity and complicating treatment approaches [71, 72]. MicroRNA-146a has recently emerged as a pivotal modulator of inflammatory pathways, with significant implications for the management of Treatment-Resistant Depression. It functions as a negative feedback regulator in controlling inflammatory signaling within key pathways, including the TLR and NF-κB pathways [72]. The regulatory role of miR-146a in neuroinflammation has been detailed earlier (see Section 2.1) and forms the mechanistic basis for its potential as a diagnostic and therapeutic tool in TRD [72, 73]. MiR-146a acts on neuroinflammatory processes in depression by repressing excessive inflammation.

Various studies have demonstrated that the overexpression of miR-146a in cell types, such as PBMCs and human corneal epithelial cells, diminishes cytokine production. For example, overexpression of miR-146a suppresses TNF-α-mediated IL-6 and IL-8 expression, whereas inhibition of miR-146a leads to elevated cytokine levels [72, 74]. Previous studies have shown that low miR-146a expression in patients with TRD is associated with high levels of TNF-α, which characterizes a pro-inflammatory state that contributes to symptom persistence by perpetuating neuroinflammation. These data suggest the therapeutic value of targeting miR-146a in TRD as an adjunctive approach for managing neuroinflammation and improving clinical outcomes [75].

The miR-146a regulatory function extends to other neuroinflammatory diseases as well. In TBI models, high levels of miR-146a have been associated with lower expression of the pro-inflammatory cytokines IL-6 and IL-1β, thus underlining a protective mechanism against injury caused by inflammation [76]. In contrast, he role of miR-146a appears to be context-dependent, as studies on advanced AD have indicated low levels of miR-146a, highlighting the complex and dynamic role of miR-146a in various stages and types of neuroinflammatory diseases [77].

In conclusion, miR-146a is a promising target for therapeutic intervention in reducing neuroinflammation in TRD, as therapeutic modulation of this miRNA might lead to more effective symptomatic management and potential improvements in treatment response owing to its key regulatory role in inflammatory pathways [78, 79].

### 2.4 Microglial Activation and Exosome-mediated Transfer of miR-146a During Neuroinflammation and Depression

Microglia are resident immune cells of the central nervous system that actively participate in the responses to stress and injury, thus driving neuroinflammatory processes implicated in the pathology of depressive disorders, including TRD. There is emerging evidence that miR-146a is a crucial anti-inflammatory miRNA involved in regulating inflammatory pathways in microglial cells [80-82]. MiR-146a plays a role in intercellular communication between microglia and neurons through exosomes, facilitating the transfer of anti-inflammatory signals [45]. The present review aims to summarize the findings concerning the role of miR-146a in these pathways and its potential therapeutic value in modulating neuroinflammation in TRD [83].

MiR-146a expression in the inflammatory pathways: miR-146a acts as a negative feedback regulator of critical inflammatory pathways, including the TLR and NF-κB signaling pathways. It directly represses NF-κB activity by targeting IRAK1 and TRAF6, which in turn decreases the expression of pro-inflammatory cytokines, such as IL-6, IL-1β, and TNF-α [57, 67, 83].

This mechanism dampens excessive inflammation, which could otherwise contribute to increased depressive symptoms and disturb the proper management of TRD. Dysregulation or low levels of miR-146a have been associated with increased pro-inflammatory conditions in patients with TRD, especially in peripheral blood mononuclear cells, where the downregulation of miR-146a corresponds to higher levels of TNF-α [84, 85]. This finding highlights the pathway through which neuroinflammation perpetuates the symptomatology of depression and suggests that miR-146a is an important regulator in depressive pathophysiology.

### 2.5 Transfer of miR-146a by Microglial Exosomes

Exosomal communication involves the extracellular vesicles of exosomes, which transfer miRNAs, proteins, and other molecules into target cells. Within the context of TRD, miR-146a-enriched exosomes released from microglia may modulate neuronal inflammation and neurogenesis, particularly in brain regions such as the hippocampus [80].

In neurons, miR-146a targets several transcription factors, including Krüppel-like factor 4 (KLF4), which is integral to neurogenesis. This suggests that exosomal miR-146a may play a dual role in fostering neuronal health while dampening neuroinflammatory responses [45].

Dual roles in inflammation and neurogenesis: The modulation of KLF4 and inflammatory cytokine expression by miR-146a plays a dual role in neuroinflammation and neurogenesis, supporting the restoration of neuronal resilience during inflammation. This dichotomy makes miR-146a a potential candidate for therapeutic approaches in TRD, wherein neuroinflammatory dysregulation and impaired neurogenesis often coexist [80, 86].

## 3 CLINICAL RELEVANCE OF MIR-146A IN TRD

miR-146a is involved in regulating neuroinflammation, and its effect on TRD makes it a promising candidate for both diagnostic and therapeutic applications. Thus, miR-146a is a potential biomarker, therapeutic target, and an adjunctive combined treatment strategy with traditional antidepressants [87].

### 3.1 Biomarker Potential

miR-146a is an active modulator of inflammatory responses and may serve as a biomarker for neuroinflammatory states in TRD. This level can be monitored during the assessments [67].

Patient Stratification: This approach would enable clinicians to position themselves to stratify patients based on their potential responsiveness to anti-inflammatory treatments by evaluating their inflammatory profiles using miR-146a. This will aid in guiding personalized therapeutic strategies in the future [67].

Disease course monitoring: Changes in the level of miR-146a may indicate the course of neuroinflammation over time, reflecting the severity of the disease course and response to treatments. High levels could reflect unresolved inflammation, whereas normalization following intervention would indicate successful treatment [88, 89].

Clinical Improvement *vs*. levels of miR-146a: A decrease in the levels of miR-146a may therefore indicate clinical improvement in patients treated with anti-inflammatory interventions and might therefore be regarded as a measurable parameter of therapeutic efficiency [45, 90].

### 3.2 Therapeutic Target

Targeting miR-146a presents a novel strategy for managing TRD by directly targeting inflammatory mechanisms [67, 72, 83, 84].

Functional Mimicry and Reconstitution: Reconstituting the levels of miR-146a or utilization of synthetic mimics downregulated the pro-inflammatory cytokines IL-6, IL-1β, and TNF-α, thereby ameliorating neuroinflammatory processes. This could reduce the symptoms of TRD associated with chronic inflammation and improve the effectiveness of conventional antidepressants [88].

Mechanistic pathways: miR-146а inhibits IRAK1 and TRAF6, which are key intermediates in TLR and NF-κB signaling pathways. As a result of modulating these pathways, miR-146a acts to put the brake on excessive cytokine production and diminished inflammation, leading to a reduced impact on depressive symptoms [91].

### 3.3 Combination Therapy Potential

MiR-146a modulation might be synergistic with conventional antidepressant treatments by virtue of its anti-inflammatory action, and would therefore offer a holistic approach:

Improved efficacy with synergistic effects: miR-146a anti-inflammation-based combination therapy may be combined with SSRIs and/or cognitive therapies for better efficacy [92]. This two-fold approach ensured that the main biochemical imbalances and inflammatory mechanisms of TRD were addressed. A holistic management strategy may involve combination therapies that include the modulation of miR-146a to comprehensively manage the activity of neurotransmitters and control inflammation in TRD [93, 94]. This integrative approach may improve symptom control and reduce relapse rates, thereby meeting the need for long-term management of TRD. It exerts an important regulatory function in neuroinflammatory pathways by modulating inflammation and neurogenesis, at least in part through mechanisms such as microglial activation and exosome transfer to neurons [95]. This duality in downregulating inflammatory responses and promoting neuronal health confers great utility to this miRNA as a biomarker and a therapeutic target for TRD. Further research is needed to elucidate the exact mechanisms and clinical utility of miR-146a, particularly in relation to its potential contribution to personalized interventions and improvements in treatment outcomes for TRD [93]. Fig. (**7**) illustrates the dopaminergic synapse, highlighting the key steps in dopamine signaling. Dysregulation of this pathway has been implicated in the mood and cognitive impairments associated with treatment-resistant depression.

### 3.4 Implications of miRNAs in TRD Treatment

Given the involvement of specific miRNAs in modulating neuroinflammation, neuroplasticity, and stress response pathways, they are exemplary candidate biomarkers and therapeutic targets in TRD. Dysregulation of miRNAs such as miR-146a, miR-124, and miR-34c, which are associated with core mechanisms underlying TRD, offers new avenues for targeted treatment strategies [51]. miR-146a has emerged as a critical regulator of neuroinflammatory pathways, where it modulates NF-κB signaling by targeting key upstream adapters such as TRAF6 and IRAK1. This regulation results in the downregulation of pro-inflammatory cytokines, including IL-6 and TNF-α, linking miR-146a dysfunction to the persistent inflammatory state observed in TRD.

MiR-146a is a key regulator in Treatment-Resistant Depression (TRD), playing a critical role in neuroinflammation. It generally acts as a negative feedback regulator of inflammatory pathways, such as the TLR and NF-κB signaling cascades [83, 88, 92]. This has been suggested to be an important mechanism for avoiding excessive inflammatory responses that might contribute to depressive symptoms [57, 67, 90].

As it influences inflammatory processes, miR-146a may serve as a biomarker for neuroinflammatory states in TRD, which would aid clinicians in stratifying patients for anti-inflammatory treatments. Overexpression or functional mimicry of miR-146a may facilitate the superior modulation of inflammation and improvement of clinical outcomes by reducing neuroinflammation and its associated symptoms [18, 83, 87]. Fig. (**8**) shows the interaction network among the miRNAs implicated in depression. It demonstrated how various miRNAs regulated each other, highlighting their contribution to neuroinflammatory processes and treatment resistance in depression.

### 3.5 miR-124 and Neuroplasticity

Neuroplasticity plays a role in the brain's ability to make structural and functional adaptations to overcome depression. miR-124 is a critical regulator of neurogenesis, synaptic plasticity, and neuronal survival [96]. Finally, miR-124 targets BDNF expression, an important modulator of synaptic strength and resilience. Chronic stress models have demonstrated that increased levels of miR-124 are associated with decreased levels of both BDNF and neuroplasticity; the latter factors increase vulnerability to depressive disorders [97].

Restoration of miR-124 levels in TRD enhances neurogenesis and facilitates synaptic function, thereby promoting recovery from depressive symptoms when neuroplasticity is compromised. These restorations have implications for antidepressant therapy, in which the regulation of miR-124 might further improve efficacy not only in traditional antidepressant treatments but also lay the groundwork for combined treatments that utilize both pharmacological and molecular pathways [98, 99].

### 3.6 miRNAs' Regulation on the HPA Axis

The hypothalamic-pituitary-adrenal axis, a component of the body's stress response, is often dysregulated in TRD, contributing to sustained elevated cortisol levels and chronic stress. Some miRNAs, such as miR-18a, miR-124, and miR-34c, interact with key components of the HPA axis to influence stress-adaptation mechanisms [100].

MiR-18a regulates the sensitivity of the glucocorticoid receptor, a crucial factor in adapting to stress. High levels of miR-124 downregulate the FKBP5-a protein, which is involved in regulating glucocorticoid receptor activity and the responsiveness of the HPA axis. MiR-34c directly affects the function of the HPA axis by targeting CRHR1, thereby influencing stress hormone sensitivity and regulating cortisol levels [101, 102]. In TRD, the pathological upregulation of miR-34c expression disrupts feedback within the HPA axis, fostering maladaptive chronic stress responses that worsen depressive symptoms [64].

Therefore, these miRNAs hold great promise not only in basic research into the mechanisms of stress response but also as therapeutic targets for normalizing the HPA axis. Modulation of miR-34c and other related miRNAs may reset stress responses, thereby breaking the cycle of complications that chronic stress complicates management [16, 103].

Therapeutic Implications and Future Directions The multilevel roles of miR-146a, miR-124, and miR-34c in TRD highlight their potential as biomarkers and therapeutic targets. These miRNAs provide pathways for intervention by modulating neuroinflammation, neuroplasticity, and stress responses, thereby addressing the core pathophysiological processes of TRD [104]. The mechanism by which these miRNAs influence neuronal pathways should be the focus of future research. Their clinical applicability as biomarkers requires further validation, and their utility as components of targeted treatment regimens ensures a promising future [105]. These miRNAs have integrated therapeutic approaches that might bridge gaps in current TRD treatments and bring hope to patients in whom conventional therapies have fallen short [106]. MiRNAs, such as miR-135a and miR-146a which have been identified as influencing these hallmark pathways hold great promise for use as panel biomarkers for diagnosis or as targets of therapy in combinatorial applications. Further studies are warranted to determine the feasibility of stratifying patients with TRD for personalized treatment.

## 4 CLINICAL BIOMARKER POTENTIAL OF MIRNAS IN TRD

- Blood and Cerebrospinal Fluid (CSF) miRNAs: Discuss the potential of peripheral miRNAs as non-invasive biomarkers for TRD and review which miRNAs are consistently altered in TRD compared to non-resistant MDD [107].
- Diagnostic *vs*. predictive biomarkers: Distinguishing between miRNAs as diagnostic markers (indicating TRD presence) and predictive markers (indicating treatment response). For example, miRNAs have been linked to antidepressant resistance and non-response to SSRI treatment [108]. The dual functions of miRNAs confer cutoffs for both the diagnostic and predictive biomarkers of TRD. Fig. (**9**) illustrates the interaction map, highlighting the complexity of the miRNA-gene relationship and demonstrating that miR-135a and miR-146a serve as the primary connecting nodes. High miR-146a levels are associated with inflammatory markers, whereas miR-135a levels predict the SSRI treatment response based on the modulation of serotonin transporter expression. This dual functionality adds value to miRNAs in psychiatric approaches for the treatment of TRD.
- Challenges in biomarker development: Addressing obstacles in translating miRNA research into clinical biomarkers, including issues of specificity, stability in biofluids, and interindividual variability [109].

### 4.1 miRNAs with Clinical Biomarker Potential in TRD

Because of their stability in blood and CSF biofluids and their ability to modulate crucial biological processes such as neuroinflammation, neuroplasticity, and cellular stress responses, miRNAs have recently emerged as promising clinical biomarkers for diagnosis and treatment response prediction in TRD [110]. The dysregulated levels of miRNAs in TRD suggest that they are non-coding RNA molecules that exert their control in the regulation of gene expression at the post-transcriptional level by binding to mRNAs and are strongly associated with the pathophysiology of diseases; thus, they represent a new, non-invasive diagnostic and prognostic tool for TRD [18].

### 4.2 Diagnostic and Predictive miRNAs: Applications in TRD

Several studies have investigated the role of miRNAs in TRD, highlighting their potential as diagnostic and predictive biomarkers.

Biomarkers of Diagnosis: Presence and Severity of TRD Diagnostic biomarkers will confirm the presence and, potentially, the severity of TRD. Some miRNAs, such as miR-146a, miR-335-5p, and miR-135a, exhibit altered expression levels in patients with TRD and, as a result, have potential as peripheral blood-based diagnostic biomarkers [7, 111].

MiR-146a, which is overexpressed in patients with TRD, is involved in the inflammatory pathways and may thus be a potential biomarker for the neuroinflammatory profile of patients with TRD. The special overexpression of this miRNA in TRD may indicate an miRNA that contributes to early diagnosis, allowing clinicians to tailor anti-inflammatory treatments [45].

miR-335-5p and miR-1292-3p: An investigation revealed dysregulated levels of these miRNAs within the TRD, with high expression levels of miR-335-5p and low expression levels of miR-1292-3p in the plasma exosomes. These findings form the basis for their application in the diagnosis of TRD and for directly relating their involvement to neurobiological pathways related to synaptic plasticity and axonogenesis [52].

miR-135a: Resistance to treatment with selective serotonin reuptake inhibitors. High miR-135a levels facilitate early diagnosis and provide clinicians with a more accurate estimate of treatment resistance. This finding underlines a better understanding of the molecular profiles of TRD [66].

These diagnostic biomarkers have the potential to allow clinicians to institute early detection and tailor treatments unique to each patient's molecular profile, thereby improving precision in the management of TRD [112].

Predictive Biomarkers: Forecasting Treatment Response in TRD. Predictive biomarkers provide crucial information on a patient's potential response to a specific mode of treatment, thereby helping clinicians make informed decisions in optimizing therapeutic strategies for TRD [113]. Examples include:

MiR-1202: Correlated with SSRI resistance, an alteration in miR-1202 levels may predict treatment outcomes by providing clinicians with clues regarding poor response to SSRIs and the need for alternative therapies [114].

MiR-187 and miR-339-5p: Both miRNAs have been identified as predictive biomarkers of non-response to SSRIs and other classes of antidepressants that provide a variable course of individualized treatment [115].

MiR-155 has been implicated in the inflammatory response in depression, with high levels in patients with TRD, perhaps presenting with resistance to conventional antidepressants [116]. This miRNA provides a pathway for inflammation-based treatment resistance assessment and may guide adjuvant anti-inflammatory therapies [116]. Therefore, these predictive biomarkers will enable clinicians to make informed decisions regarding suitable treatment regimens, thereby enhancing the precision of interventions and potentially improving patient outcomes by targeting individual biochemical profiles [116].

The utility of miRNAs as biomarkers in blood-derived biofluids is more accessible. It thus allows for convenient, non-invasive assessment compared to the more invasive CSF collection, which offers higher specificity for CNS pathologies [117]. For example, miR-34a is elevated in the CSF and is associated with neurodegenerative and synaptic dysfunction, whereas miR-9 is associated with neuroplasticity and mood regulation in TRD [117].

These findings justify the use of CSF-derived miRNAs to obtain detailed insights into the CNS-specific pathologies in TRD. Meanwhile, blood-based miRNAs such as miR-146a and miR-155 represent feasible options for routine clinical monitoring of the disease course and response to treatment [118].

Summary and clinical implications: Collectively, these studies highlight the potential of miRNAs as diagnostic and predictive biomarkers for TRD. Diagnostic miRNAs, such as miR-146a and miR-335-5p, are capable of early identification of TRD and facilitate tailored treatment approaches. In contrast, predictive miRNAs, including miR-1202 and miR-155, provide insight into plausible responses to treatments [119]. Thus, the clinical practice of employing miRNA profiles promises to enhance early detection, enable precision medicine approaches, and support more personalized treatment options for patients with TRD, for whom conventional therapies have often been insufficient, as shown in Table **5**. These biomarkers should be further validated in larger cohorts, while their integration into routine clinical diagnostics and personalized care in the management of TRD is supported [120-122].

### 4.3 Biomarker Development Challenges

In this regard, some important issues must be addressed before miRNAs can be clinically applied as biomarkers for TRD. However, many miRNAs participate in various biological processes, making it challenging to associate specific miRNAs exclusively with TRD [123]. For example, miRNAs implicated in inflammation may also be dysregulated in other psychiatric and medical conditions, making them less specific as biomarkers for TRD. Overcoming this issue requires extensive research to identify the miRNAs associated with TRD and their distinct molecular pathways.

### 4.4 Stability in Biofluids

Although miRNAs are relatively stable in biofluids, their integrity may be compromised by factors such as sample handling, processing, and storage conditions [124]. There is a need for harmonized protocols for sample collection, storage, and analysis to ensure accurate quantification of biomarkers. Standardization of methodologies will ensure a lack of pre-analytical variability, which could otherwise compromise the integrity of miRNA and, hence, their reliability as biomarkers [108].

### 4.5 Inter-Individual Variation

Genetic and environmental factors, such as age, sex, lifestyle, and comorbidities, may lead to individual variations in miRNA expression levels [108]. Such variability complicates the establishment of standardized reference ranges and may be one reason why personalized baselines are necessary to interpret miRNA levels in clinical practice accurately. Individual reference points highlight the complexity of translating miRNA research into standardized clinical biomarkers [125].

### 4.6 Longitudinal Studies and Validations

Extensive validation studies are required to establish the utility of miRNA biomarkers in a diverse population, as shown in Table **6**. Longitudinal studies that follow changes in miRNA expression over time in response to treatment will eventually provide predictive and diagnostic values. This will ensure the efficacy, reliability, and reproducibility of miRNA biomarkers in the clinical setting [124, 126].

### 4.7 Comparison of Blood and CSF miRNAs for TRD

There are different advantages and disadvantages of blood miRNAs compared to miRNAs in the CSF. Blood miRNAs are easily accessible and favorable for non-invasive monitoring; however, their levels may be modulated by systemic diseases, which can reduce specificity for TRD [134, 135].

For example, blood samples have demonstrated a wide variation in detectable miRNAs, including miR-590-5p and miR-142-5p, which are associated with neurobiological processes pertinent to TRD and may thus serve as an indirect biomarker of CNS inflammation [136]. In contrast, CSF miRNAs more directly reflect the CNS physiology and pathology. The isolation of CSF miRNAs, such as miR-146a and miR-34a, provides specific insights into the neuroinflammation and neurodegeneration processes that are closely linked. However, the invasiveness of this collection method limits the feasibility of using CSF for routine clinical use. Thus, careful consideration of when and how to use CSF-based miRNA biomarkers is required for their effective use [137]. Targeting specific miRNAs for therapeutic interventions of TRD opens a new direction in understanding the mechanisms underlying the disorder. For example, miR-590-5p modulates the TGF-β pathway as a critical pathway in neuroinflammation and neuronal health, thereby providing a novel target for intervention [138, 139]. MiR-590-5p directly targets the 3' untranslated region of TGF-β RII and modulates inflammation-neuronal balance. In TRD, neuroinflammation [140, 141].

Exacerbation by an overactive TGF-β pathway may lead to increased depressive symptoms. These imbalances in the TGF-β signaling pathway may be restored by treatments that modulate miR-590-5p levels [142]. The investigation of miRNAs as biomarkers in TRD highlights the potential to identify specific miRNAs, such as miR-146a, miR-34a, and miR-9, in the blood and CSF, which could fully revolutionize the diagnostic and predictive capabilities of noninvasive and precise biomarkers for TRD [143]. However, specificity, biofluid stability, inter-individual variability, and most importantly, validation, remain significant challenges for clinical translation. This will need to be overcome through rigorous research, standardized protocols, and longitudinal validation to realize the full potential of miRNAs in the clinical realm. Ultimately, miRNA biomarkers may provide new therapies targeting specific mechanisms and improving disease management by offering insights into the molecular underpinnings of the disorder [110, 144].

## 5 THERAPEUTIC POTENTIAL OF TARGETING MIRNAS IN TRD

### 5.1 Therapeutic Potential of Targeting miRNAs in TRD

In addition to diagnostics, miRNAs provide new therapeutic opportunities for TRD. This mainly involves the use of miRNA mimics and antagomiRs, which restore and inhibit miRNA function, respectively [103]. For instance, miRNAs mimic miR-124 to enhance neurogenesis by upregulating neurotrophic factors, whereas antagomiRs of miR-155 reduce neuroinflammation by silencing inflammatory pathways [98, 145]. This therapeutic approach holds the most promise because it can target multiple pathways in parallel, thereby satisfying the multivariate nature of the TRD pathology. Such miRNA-based interventions can be combined with other currently used treatments, such as SSRIs or ECT, to achieve enhanced therapeutic efficacy through synergistic mechanisms [146, 147].

Treatment-resistant depression remains a major therapeutic challenge in psychiatry, with many patients being unresponsive to conventional antidepressant therapies. MicroRNAs are small, non-coding RNA molecules that have a regulatory function at the gene expression level and have emerged as both potential therapeutic targets and biomarkers in TRD [6]. This section discusses miRNA-based therapeutic strategies that may potentially improve the efficacy of antidepressant treatments, along with their ethical and practical considerations [7]. Fig. (**10**) shows that miRNA-pathway interactions are interconnected, allowing the possibility of various pathway combination therapies. Examples include miR-146a and miR-34c, which affect the neuroinflammatory and stress pathways, respectively, and thus show potential for synergistic interactions with psychotherapy or ECT. For example, modulation of miR-34c may be used to potentiate normalization of the HPA axis, while targeting miR-146a can reduce inflammation-induced neurodegeneration. These findings suggest a method for developing integrative therapeutic strategies for TRD.

### 5.2 miRNA-based Therapeutics

miRNAs play a critical role in regulating gene expression by binding to target mRNAs, leading to their degradation or inhibition of translation [51, 148]. In TRD, dysregulation of specific miRNAs can lead to persistence of symptoms and resistance to therapy. Regarding therapeutic aims, two approaches are currently being investigated [51]. MiRNA mimics and antagomiRs. miRNAs: Synthetic molecules and miRNA mimics are designed to mimic the function of endogenous miRNAs and those downregulated in TRD. Therefore, mimics tend to restore normal regula-tory functions by reintroducing these miRNAs and correcting aberrant gene expression associated with depressive pathologies. For example, miR-124, which targets neurogenesis and synaptic plasticity, is highly expressed in patients with TRD, suggesting its potential to reverse treatment resistance [149-151].

Increasing miR-124 levels using mimics may enhance neuroplasticity, precipitating a resilient response to stress, and exerting a therapeutic effect in TRD [152]. AntagomiRs are chemotherapy-engineered oligonucleotides that inhibit specific miRNAs overexpressed in TRD by releasing their suppressive effects on the target mRNAs [153]. For example, antagomiRs directed against miR-155 represent a promising strategy, because miR-155 is deeply involved in neuroinflammation, a process often related to depressive disorders. Downregulation of pro-inflammatory cytokine expression by miR-155-targeting antagomiRs could help reduce the inflammatory states that contribute to TRD [70].

Despite their therapeutic promise, miRNA-based interventions face several critical limitations. One key challenge is the inherent instability of miRNAs in circulation, particularly in unprotected forms, where nucleases rapidly degrade them. This raises concerns about their consistency as biomarkers and the efficiency of delivery in therapeutic applications. Additionally, due to their ability to target multiple mRNAs, miRNAs carry a substantial risk of off-target effects, potentially altering gene expression networks beyond the intended pathway. These unintended interactions may complicate therapeutic outcomes or induce secondary pathologies. From a translational perspective, regulatory hurdles remain considerable. Currently, there are no standardized protocols governing the development of miRNA therapeutics, and limited clinical trial data are available to support their safety, efficacy, and long-term tolerability. As a result, the pathway to clinical approval remains uncertain and requires further harmonization across regulatory frameworks.

### 5.3 miRNA Modulation for Enhancement of Antidepressant Response

Targeting specific miRNAs may offer hope for enhanced responsiveness to current antidepressant therapies, especially in cases of treatment-resistant depression when classic treatments are ineffective [154]. Several miRNAs have been identified as key modulators of neurotransmitter pathways and thus may have the potential to enhance the effects of antidepressants [155]. miR-135a controls serotonin transporter and receptor signaling. The knockdown of miR-135a has been shown to increase serotonin levels, potentially enhancing the efficacy of SSRIs. As it is an inhibitor, miR-135a mimics are an active adjuvant therapy to improve outcomes in patients resistant to SSRIs [66]. The expression of miR-1202 has been associated with non-response to SSRIs; hence, it may be considered as a predictive biomarker of antidepressant treatment response. Monitoring the expression levels of miR-1202 may enable clinicians to more accurately predict the likelihood of SSRI responsiveness in patients, thereby allowing them to optimize their treatment strategies and reduce the unnecessary trial-and-error associated with antidepressant therapies [156, 157].

### 5.4 Ethical and Practical Considerations

Notwithstanding the encouraging potential of miRNA-based interventions in treating TRD, several challenges and ethical considerations must be addressed before translating these therapies into safe clinical practice [149]. The BBB remains a formidable barrier for the delivery of miRNA-based therapies to the central nervous system. Some of the pathway options explored for crossing the BBB *via* miRNA therapeutic transport include nanoparticle-based delivery systems and viral vectors [158]. However, the success of these interventions is contingent upon their effective and selective delivery into brain tissues without any harmful side effects. Long-term safety and off-target effects: Chronic manipulation of miRNA expression may lead to unintended changes in gene expression [159]. Given that miRNAs typically have numerous target genes, off-target effects may pose a significant threat and can even exacerbate existing conditions or induce new side effects. Such therapies require rigorous preclinical and clinical testing for safety, with the major objective being to reduce off-target interactions [160]. One of the major challenges in miRNA therapeutics is the efficient delivery of miRNA mimics or inhibitors to the central nervous system. Several strategies utilizing nanoparticle-based delivery systems or viral vectors have been investigated to enhance specificity and facilitate the crossing of the blood-brain barrier [161].

Ethical considerations regarding the use of genetic and molecular interventions raise concerns about the potential long-term effects that may impact cognition and behavior [162]. Special attention should be paid to informed consent, patient autonomy, and societal acceptance. Interactions with bioethicists, patients, and the public are necessary to introduce miRNA-based therapies responsibly and ethically [163].

The therapeutic utility of miRNAs in treatment-resistant depression opens new and promising avenues in psychiatry. The application of miRNA mimics or antagomiRs may enable more personalized and effective treatments by restoring normal brain function and enhancing the efficacy of antidepressants [51]. However, successful clinical translation is impeded by substantial hurdles related to the delivery systems, long-term safety, and off-target effects. Further interdisciplinary research and collaboration are necessary to fully harness the therapeutic potential of miRNAs in TRD, with the goal of enhancing patient outcomes in this debilitating condition [164].

## 6 FUTURE DIRECTIONS IN MIRNA RESEARCH FOR TRD

The promise of miRNA interventions in TRD treatment has encouraged various lines of investigation, with the potential to revolutionize our understanding and treatment of this multifaceted illness [51]. Some of the proposed directions for refining miRNA-based approaches in therapy are as follows:

### 6.1 Individualized miRNA Profiles for TRD

Individual miRNA profiles can greatly enhance the precision of TRD treatments. Based on genetic predispositions and environmental factors such as the history of stressful stimuli experienced by a patient, researchers can create personalized profiles with more precise therapeutic options. Personalization was performed using the double approach [51]. Using high-throughput sequencing, it is possible to connect genetic variations, such as SNPs, to specific miRNA expression patterns in patients with TRD. These findings provide recommendations for future research. For instance, exosomal miRNAs sequenced in plasma across different cohorts may express a pattern indicative of TRD that can be directly treated at the individual level [165].

Further developments may come from integrating psychosocial data, such as the history of trauma, into miRNA profiles. Longitudinal studies could then correlate exposure to stress with fluctuations in miRNA levels to tailor interventions to a specific patient experience [166]. Such personalized profiles may form the basis for developing precision medicine approaches that improve treatment outcomes by incorporating the unique biological and psychosocial contexts of each patient [167].

### 6.2 Combination Therapies with miRNA Modulation

Consequently, the combination of miRNA modulation with other established treatment modalities, including electroconvulsive therapy and psychotherapy, is a promising avenue for addressing the multifaceted nature of TRD [168]. The use of this combination is observed in the following cases.

ECT may be used to induce neurobiological changes by altering the levels of miRNAs related to neuroplasticity, such as miRNA-targeting interventions that may have additive effects when combined with ECT. Preconditioning with miRNA mimics or inhibitors can potentiate the effects of ECT by improving neuroplasticity or modulating inflammatory responses [169].

This approach may be integrated with psychotherapy, targeting the miRNAs that play a role in neuroplasticity and mood regulation, in conjunction with psychotherapeutic interventions, to optimize both biological and psychological responses. For example, the therapeutic effect of psychotherapy can be enhanced by miRNAs that target inflammatory pathways, further improving symptom management and resilience in patients with TRD [132, 170]. Combining miRNA modulation with more conventional therapies may provide additive or synergistic effects, thereby enhancing the overall treatment effectiveness and allowing more complete management of TRD [171, 172].

Fig. (**11**) presents a cytoscape-generated network of gene-gene interactions, which identifies key genetic regulators important in neuroinflammatory signaling. This graph represents the interconnected nature of genes that contribute to various dysregulated immune responses during treatment resistance.

Treatment-resistant depression is a pathophysiology involving interactions between several genes and pathways implicated in neuroinflammation, neuroplasticity, and cell survival, as shown in Fig. (**12**). A Protein-Protein Interaction (PPI) network was constructed for genes implicated in Treatment-Resistant Depression (TRD). This network identified significant hubs, such as PTK2, IL6, and BDNF, which play vital roles in key biological processes, including inflammation and synaptic plasticity. PTK2, also known as focal adhesion kinase, integrates signaling pathways that may mediate cellular responses to stress and synaptic remodeling. IL6 is a good example of a pro-inflammatory cytokine that plays a role in neuroinflammation in TRD. BDNF is recognized as a critical regulator of neuroplasticity/neuronal survival and is usually downregulated in TRD. This network further encompasses genes such as CREB1, which regulates the transcriptional activity associated with neurogenesis, and BCL2, which controls the apoptotic pathways. miRNAs target these hubs, thereby affecting their expression and subsequent signaling. For instance, miR-146a affects IL6-related inflammatory pathways, whereas miR-135a targets BDNF-related pathways, suggesting that these miRNAs are involved in pathogenesis. The dense interconnections within the network highlight the systemic nature of TRD and the therapeutic potential of targeting miRNAs to modulate the key pathways.

### 6.3 Longitudinal Studies on miRNA Expression in TRD

Longitudinal studies of miRNA expression in TRD and during treatment are necessary to attribute specific miRNAs to the phenomenon of treatment resistance. This could be achieved through the following procedures:

Repeated biomarker assessments: Regular monitoring of the levels of the relevant miRNAs in the blood or CSF will be used to study changes in their expression that may be associated with treatment response or resistance. For example, monitoring miRNAs such as miR-135a, which is implicated in antidepressant resistance, could clarify its role in TRD [124]. Animal model studies have suggested that miR-135a enhances serotonergic signaling and that the administration of miR-135a mimics results in reduced depression-like behaviors. These findings suggest that miR-135a may be a therapeutic target for enhancing antidepressant efficacy, particularly in patients [66, 173].

Clinical Outcome Correlation: A study would thus be in a position to identify any trend that may correspond to symptomatic changes in patients in relation to variations in miRNA levels, thereby predicting treatment effectiveness [103].

This would not only emphasize which miRNAs are directly involved in TRD but also suggest the optimal timing of intervention. This could imply the development of predictive biomarkers based on anticipating responses to various treatments, thus allowing a dynamic and responsive approach to managing TRD [16].

### 6.4 Meeting miRNA-Specific Challenges in TRD Therapy

Although miRNA-based therapies have much to offer, several challenges must be overcome before they can be applied clinically.

Too many targets for the miRNA effect (TMTME): One crucial problem is the fact that one miRNA will bind to numerous genes and may have many side effects by simultaneously interrupting several biological pathways. Approaches to enhance miRNA target specificity are vital for reducing side effects and ensuring direct effects [174].

Immune-related adverse events are a common issue in miRNA mimic clinical trials, and MRX34 has not become an exception. It also raises concerns regarding interactions with the immune system. The extra risks could thus probably be lessened by optimal dosing and delivery methods of miRNA, providing for its application in a safer manner [103]. A major challenge in delivering miRNA therapies across the BBB is the need for sufficiently non-invasive approaches. Consequently, highly developed nanoparticle-based and viral vectors may be versatile for improving miRNA transport across the BBB into the CNS. However, further validation is essential [107, 134, 158]. Recent advances in delivery system engineering have demonstrated the potential of ligand-conjugated nanoparticles to enhance brain-specific uptake of miRNA cargo. For example, transferrin-modified PLGA nanoparticles have successfully delivered miR-124 mimics to hippocampal neurons in rodent models, improving both behavioral and molecular outcomes in stress-induced depression [175, 176]. Additionally, engineered exosomes, due to their innate biocompatibility and ability to cross the BBB, have emerged as promising carriers. Preclinical studies have demonstrated that the exosome-mediated delivery of miR-132 and miR-146a to the brain attenuates neuroinflammation and enhances synaptic plasticity in chronic stress models [177]. These approaches demonstrate the increasing feasibility of targeted CNS delivery, although further optimization and clinical validation are still necessary.

Personalized profiling, combination therapies, and longitudinal tracking are various future directions in miRNA research for TRD, all of which hold strong promise for progress in the management of TRD [103, 140]. The application of these strategies will offer more detailed information on the role of miRNAs in TRD and will facilitate the development of new, personalized, and potent therapeutic approaches. Although miRNA-based approaches are still evolving, they represent a significant step toward altering the therapeutic landscape of TRD and offer new hope to patients resistant to conventional therapies [38, 173]. However, several challenges remain in miRNA-based therapies, including off-target effects, variability in miRNA expression within diverse patient groups, and the development of an efficient delivery system that can cross the blood-brain barrier. Future studies should focus on the longitudinal validation of miRNA biomarkers across diverse patient cohorts and optimize delivery platforms for miRNA-based interventions. Only these are essential for translating preclinical promise into clinical feasibility in the management of TRD.

### LIMITATIONS AND FUTURE DIRECTIONS

While this review integrates current findings on miRNA involvement in treatment-resistant depression, several limitations should be acknowledged. First, the majority of cited studies are preclinical or limited to small patient cohorts, which restricts the generalizability of findings across broader clinical populations. Second, variations in miRNA detection platforms, normalization strategies, and sample types (plasma *vs*. CSF *vs*. exosomes) complicate direct comparison between studies and may contribute to inconsistent results. Third, while mechanistic insights were highlighted, causality cannot be inferred solely from association data. Moreover, sex-based and age-specific differences in miRNA expression have not been consistently reported, which limits interpretation across demographic subgroups. Ultimately, the clinical translation of miRNA modulation remains speculative in the absence of longitudinal human trials that confirm its stability, specificity, and therapeutic response. These factors underscore the need for harmonized protocols and multicenter validation efforts in the future.

## CONCLUSION

While miRNAs offer new horizons for improving our understanding and treatment of TRD, these small non-coding RNA molecules have promising functions as critical regulators of gene expression in neuroinflammation, neuroplasticity, and stress response pathways, which are implicated in the pathophysiology of TRD. Their potential as biomarkers is strongly compelling, not only for diagnosis but also for monitoring treatment response, thereby opening new avenues for more personalized psychiatric care.

The utility of miRNAs as biomarkers lies at the core of precision psychiatry, as they enable individualized profiles, leading to targeted and effective therapies. Moreover, therapeutic strategies targeting the modulation of miRNAs will open new avenues for treating the biological complexity of TRD using miRNA mimics and antagomiRs. Thus, it is possible to enhance responses to classic treatments, such as selective serotonin reuptake inhibitors, electroconvulsive therapy, and psychotherapy.

Several important barriers must be overcome before miRNA-based interventions become more widely accepted. Most of these involve effective delivery across the blood-brain barrier, evasion of immune-mediated adverse effects, and enhanced specificity to reduce off-target effects. The full therapeutic potential of miRNAs cannot be harnessed in clinical settings without addressing these issues to ensure their safety and efficacy.

Among the miRNAs reviewed, several stand out for their potential clinical translation in treating treatment-resistant depression. miR-146a is the most prominent candidate due to its anti-inflammatory activity *via* the NF-κB pathway and its dual role as a biomarker and therapeutic target. miR-135a shows promise in modulating serotonin transporter expression, positioning it as a predictive biomarker for SSRI response. miR-124, implicated in neurogenesis and synaptic remodeling, has therapeutic value in restoring neuroplasticity. miR-155, consistently elevated in TRD, contributes to chronic inflammation and may guide anti-inflammatory interventions. These molecules are supported by converging mechanistic, diagnostic, and translational findings.

While miRNAs offer a promising path forward in precision psychiatry, several barriers still need to be addressed. These include stability in biofluids, targeted delivery across the blood-brain barrier, minimization of off-target effects, and adherence to regulatory standards. Continued validation through longitudinal human studies, along with improved delivery platforms, will be essential for advancing miRNA-based interventions from experimental models to clinical settings.

## Figures and Tables

**Fig. (1):**
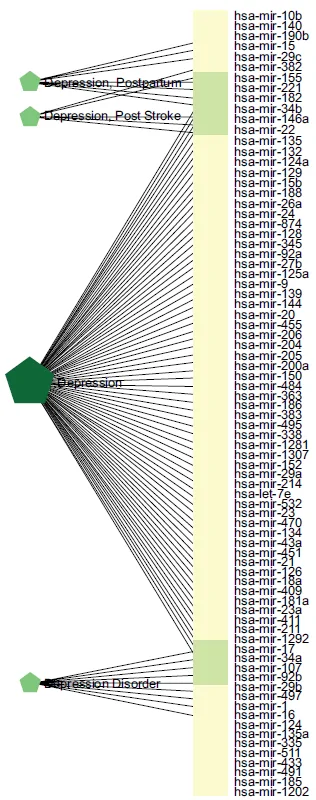
Interaction network of depression-related miRNAs across diagnostic subtypes. This figure maps the regulatory interactions between key microRNAs and depression phenotypes, including major depressive disorder, postpartum depression, and post-stroke depression. Nodes represent specific miRNAs, while edges denote experimentally validated or computationally predicted associations with subtype-specific pathways. The network illustrates the central regulatory influence of miRNAs such as miR-124 and miR-146a across multiple inflammatory and neuroplasticity-linked mechanisms. These shared and divergent regulatory patterns highlight the molecular heterogeneity of depressive disorders and suggest miRNA-based stratification strategies for clinical application.

**Fig. (2):**
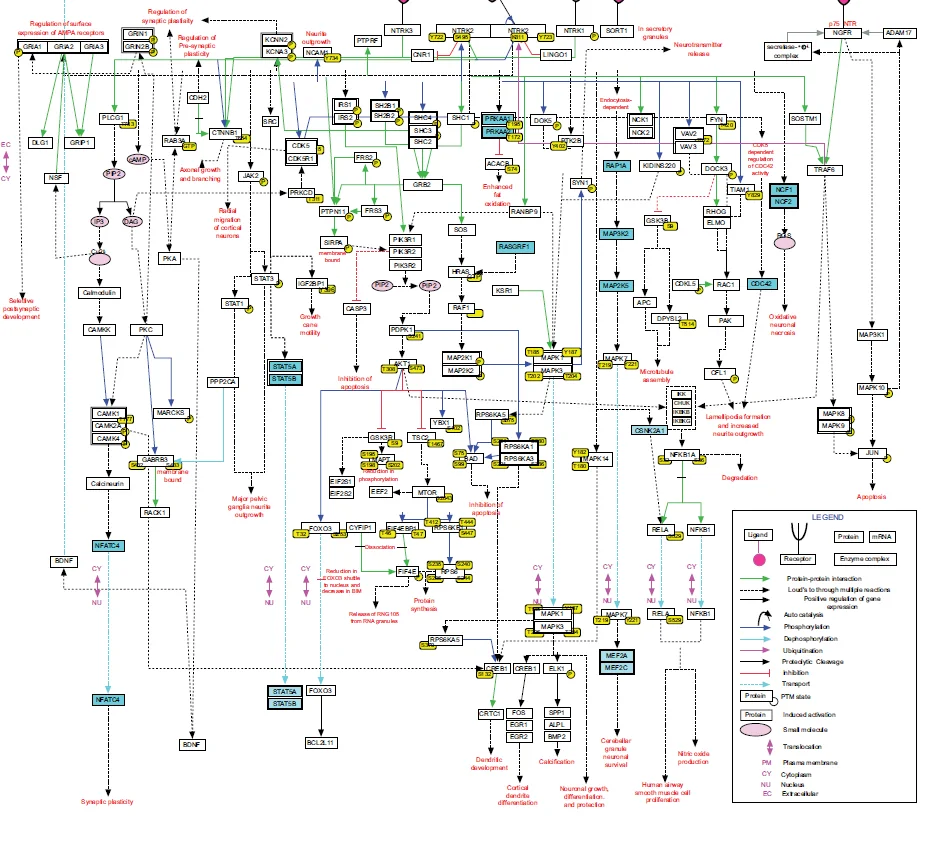
Brain-Derived Neurotrophic Factor (BDNF) signaling pathway and its disruption in TRD. The schematic illustrates the BDNF–TrkB signaling cascade, highlighting the downstream pathways crucial for synaptic plasticity, dendritic remodeling, and neuronal survival. In TRD, downregulation of BDNF leads to impaired activation of MAPK/ERK, PI3K/AKT, and PLCγ pathways, resulting in decreased synaptic strength and structural resilience. The figure outlines how this signaling disruption contributes to treatment resistance by reducing the brain’s adaptive response to stress and antidepressant interventions.

**Fig. (3):**
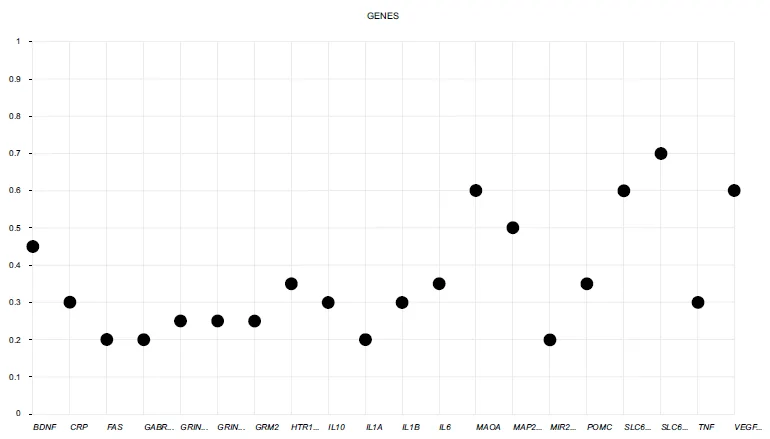
Differential gene expression in TRD: BDNF, IL-6, and TNF. This scatterplot shows the expression levels of three genes critical to TRD pathophysiology in patient samples: BDNF (neuroplasticity), IL-6 and TNF (neuroinflammation). Downregulation of BDNF correlates with cognitive and emotional dysfunction, while elevated IL-6 and TNF levels reflect persistent inflammation. These patterns support the use of these markers in diagnostic profiling and point toward inflammation-targeted adjunctive therapies.

**Fig. (4):**
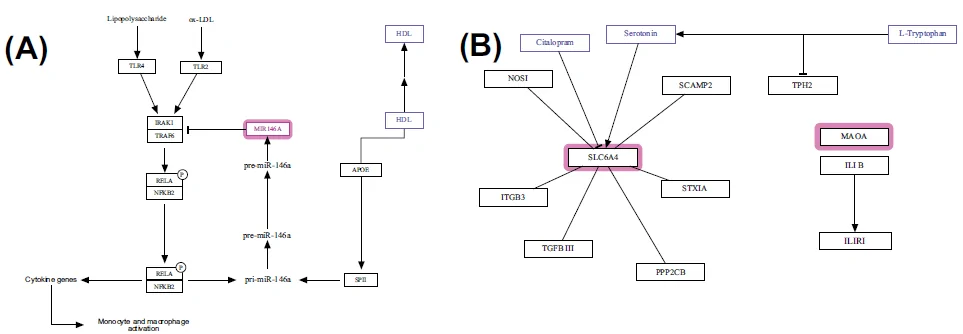
Regulatory influence of miRNAs on inflammation and serotonin signaling in TRD. (**A**) Diagram of miR-146a-mediated inhibition of NF-κB signaling through direct suppression of TRAF6 and IRAK1, leading to decreased IL-6 and TNF-α production. This pathway is central to miR-146a’s anti-inflammatory effect and its role as a biomarker in TRD. (**B**) miRNA regulation of serotonergic signaling. miR-135a targets SLC6A4 (serotonin transporter gene), influencing serotonin reuptake, while additional miRNAs modulate monoamine oxidase A (MAOA), altering serotonin catabolism. These interactions underpin miRNA-linked antidepressant responsiveness and treatment resistance.

**Fig. (5):**
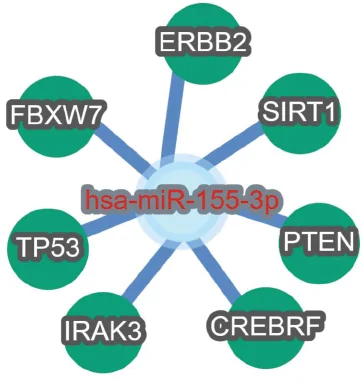
Target network of miR-155-3p in TRD. This figure illustrates the interaction between miR-155-3p and its target genes: TP53, PTEN, SIRT1, and ERBB2. These genes are involved in apoptosis, neuroplasticity, and inflammation all of which are disrupted in TRD.

**Fig. (6):**
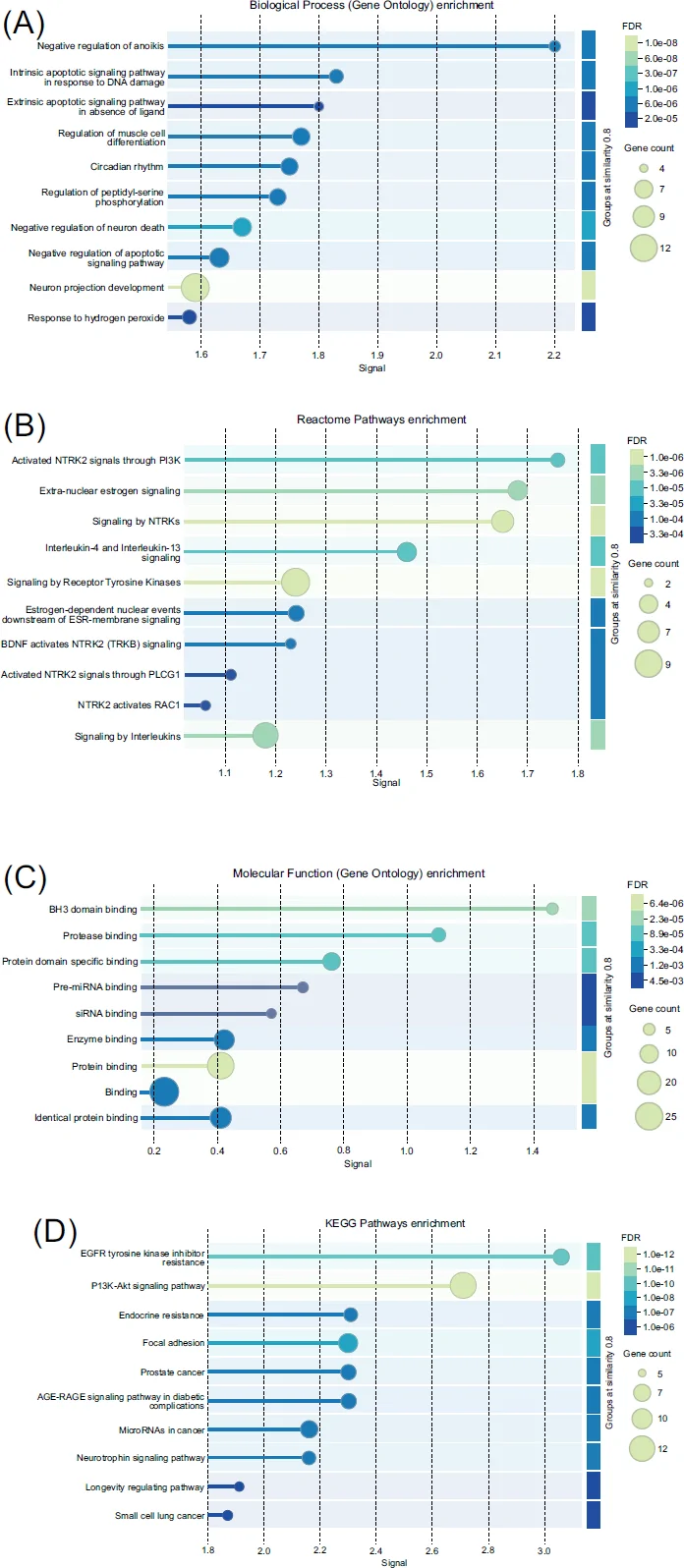
KEGG pathway analysis: miRNAs in inflammatory and serotonergic signaling relevant to TRD. (**A**) Enrichment map of KEGG pathways regulated by miR-146a, showing suppression of cytokine signaling *via* NF-κB and TLR pathways. (**B**) Pathway network regulated by miR-124, highlighting its impact on neuroinflammation and synaptic function. (**C**) Integration of miRNA targets in serotonin signaling cascades, illustrating how miR-135a and others influence reuptake and receptor sensitivity. (**D**) Combined KEGG map overlay showing the convergence of inflammatory and neurotransmitter pathways regulated by multiple miRNAs in TRD.

**Fig. (7):**
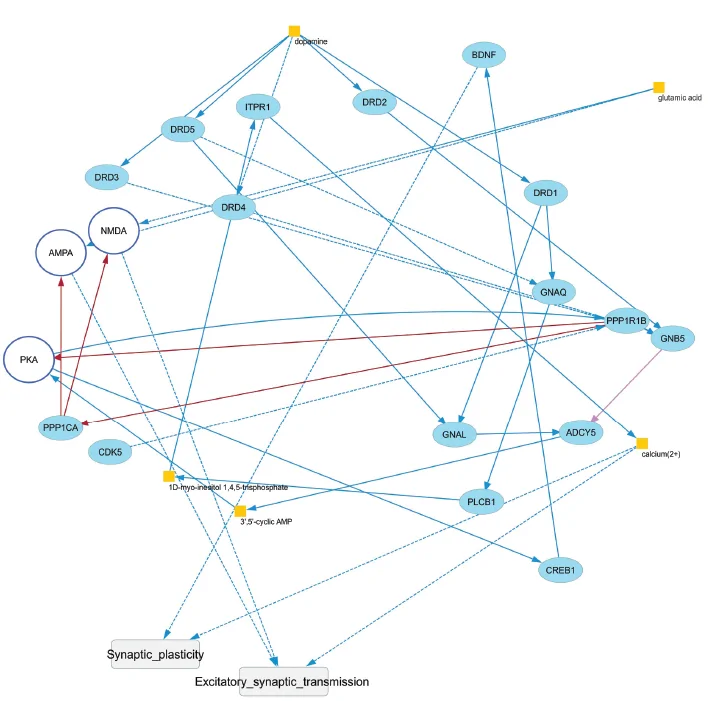
Dopaminergic synapse signaling and its dysregulation in Treatment-Resistant Depression (TRD). This diagram maps the key elements of dopaminergic neurotransmission, including dopamine synthesis, vesicular storage (*via* VMAT2), synaptic release, receptor binding (D1–D5), and reuptake through the dopamine transporter (DAT). In TRD, dysregulation is commonly observed at the level of D2 receptor signaling and DAT function, leading to impaired synaptic dopamine availability. These disruptions are associated with reduced motivation, anhedonia, and cognitive blunting, which are hallmark features of TRD. The figure also indicates modulatory influences from intracellular signaling cascades (*e.g*., cAMP/PKA) that are downstream of receptor activation. TRD-associated deficits in this pathway may underlie poor response to conventional antidepressants and are potential targets for neuromodulatory or dopaminergic augmentation therapies.

**Fig. (8):**
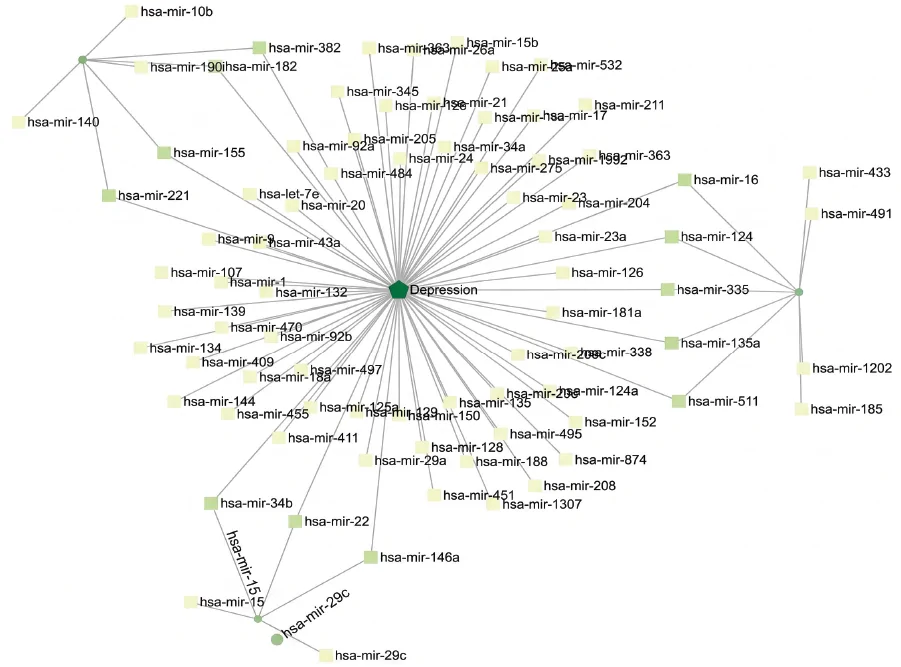
miRNA regulatory network in treatment-resistant depression. This interaction network highlights key regulatory relationships among miRNAs implicated in TRD, including miR-124, miR-146a, and miR-155. Nodes represent individual miRNAs, with edges denoting documented interactions or co-regulatory mechanisms across neuroinflammation and neuroplasticity pathways. Central miRNAs act as regulatory hubs, influencing multiple downstream processes, including cytokine release, synaptic remodeling, and glucocorticoid sensitivity. The network demonstrates how cooperative or competing regulatory actions by miRNAs contribute to the pathophysiological complexity of TRD.

**Fig. (9):**
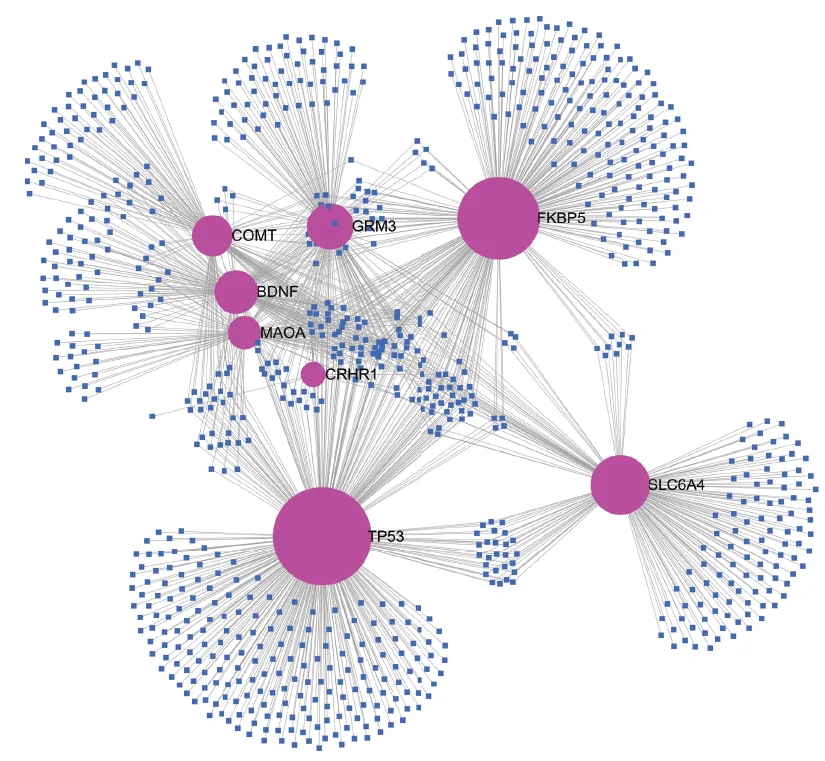
miRNet visualization of miRNA-gene interactions in TRD. This figure presents a miRNet-based interaction map showing the connections between clinically significant miRNAs (*e.g*., miR-135a, miR-146a) and their validated gene targets. The centrality of miR-135a in regulating serotonin transport (SLC6A4) and miR-146a in modulating inflammatory mediators (TRAF6, IL6) is illustrated. Each edge reflects a direct regulatory association derived from curated databases, highlighting the role of these miRNAs as potential biomarkers and therapeutic candidates in personalized TRD management.

**Fig. (10):**
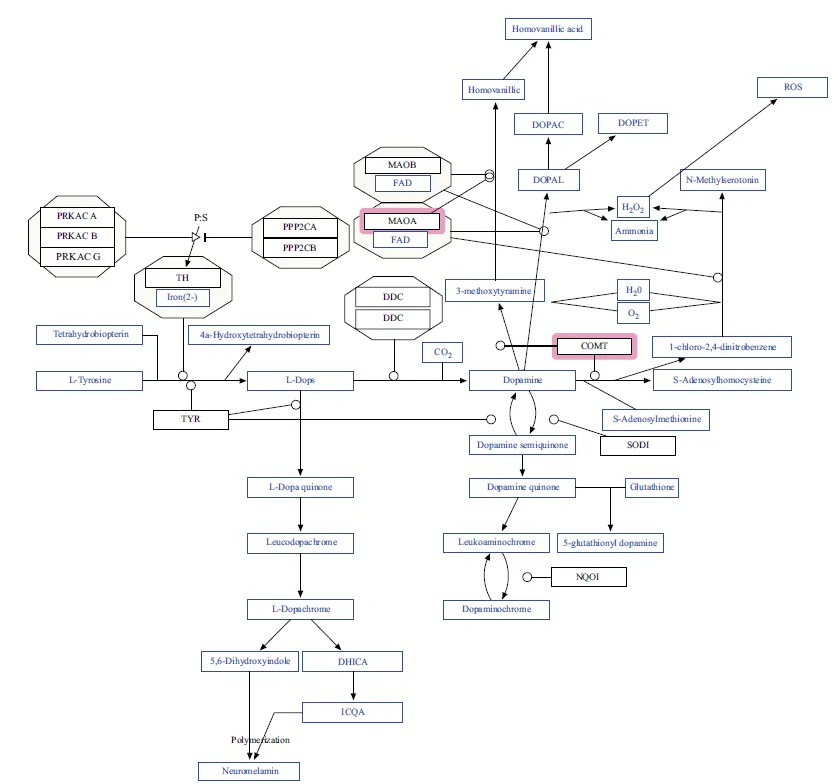
Schematic of miRNA-mediated modulation of serotonin and neuroplasticity pathways in TRD. This integrative diagram illustrates the roles of multiple miRNAs, including miR-135a, miR-124, and miR-34a, in key signaling pathways relevant to TRD. miR-135a targets the expression of the serotonin transporter and indirectly enhances synaptic serotonin levels. miR-124 modulates neurotrophic support by regulating BDNF expression, while miR-34a influences dendritic spine architecture and plasticity. The combined effect of these miRNAs contributes to antidepressant responsiveness and synaptic adaptability. This schematic emphasizes the therapeutic potential of correcting miRNA dysregulation to restore both neurotransmitter balance and neurostructural integrity.

**Fig. (11):**
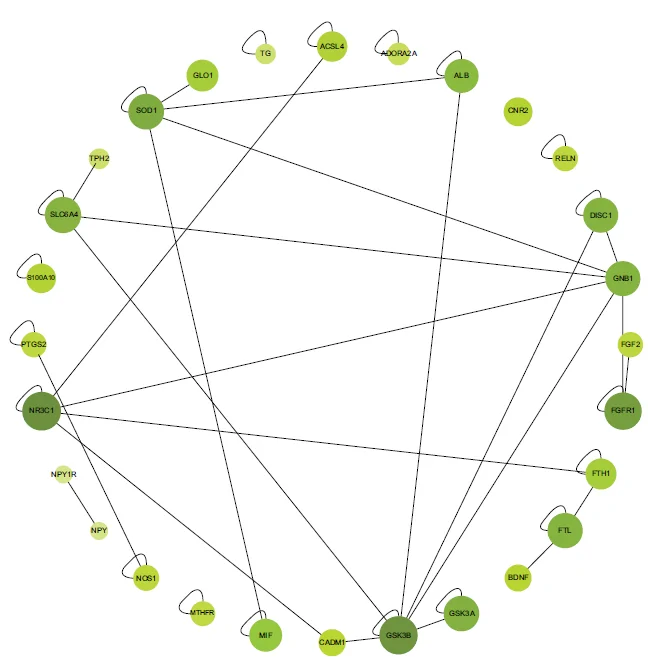
Cytoscape-generated gene–gene interaction network involved in TRD pathogenesis. The figure displays a gene–gene interaction map built using Cytoscape, focusing on genes affected by miRNA dysregulation in TRD. Nodes represent core genes implicated in neuroplasticity (*e.g*., BDNF), neuroinflammation (*e.g*., IL6, TNF), and cellular stress responses (*e.g*., TP53, PTK2). Hub genes are identified based on connectivity and interaction density, underscoring their roles as convergence points in TRD-relevant signaling pathways. This network provides a systems-level view of how miRNA-regulated targets integrate into broader pathogenic circuits.

**Fig. (12):**
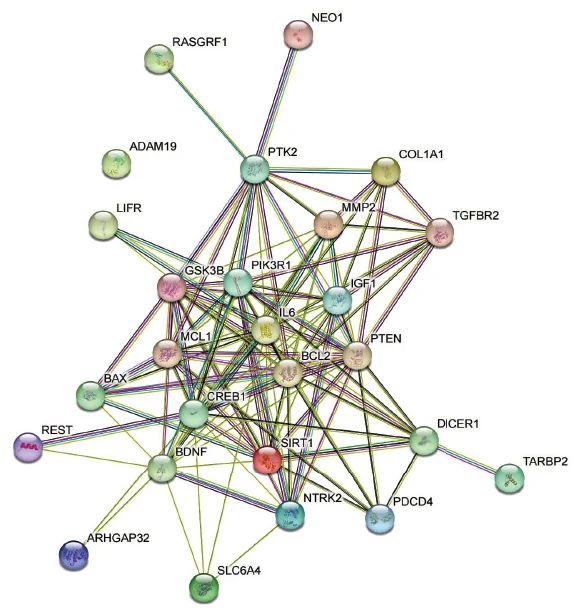
Protein–protein interaction (PPI) network of miRNA-targeted genes in TRD. This PPI network maps functional interactions among proteins encoded by genes targeted by miRNAs dysregulated in TRD. Key clusters include neurotrophic factors (*e.g*., BDNF), inflammatory mediators (*e.g*., IL6), and apoptosis regulators (*e.g*., BCL2, SIRT1). Edges indicate experimentally validated or predicted protein interactions. Central hubs in the network represent mechanistic bottlenecks whose disruption may drive TRD symptomatology. The network underscores the functional impact of miRNA dysregulation on protein-level interactions, suggesting key points for therapeutic modulation.

**Table 1:** Overview of biomarkers implicated in neuroinflammation and TRD, including functional roles, clinical relevance, and therapeutic potential.

Category	Biomarker	Role in TRD	Clinical Utility
Cytokines	IL-6	Elevated levels correlate with severity and resistance to antidepressants.	Potential stratifier for anti-inflammatory treatments; targeted by IL-6 inhibitors (*e.g*., tocilizumab).
-	TNF-α	Drives microglial activation and synaptic dysfunction.	Marker for response to TNF-α inhibitors (*e.g*., infliximab).
-	IL-1β	Activates inflammasomes, leading to neuroinflammation and dysregulation of the HPA axis.	Targeted by IL-1β antagonists (*e.g*., anakinra).
Acute-Phase Proteins	C-Reactive Protein (CRP)	Systemic marker of inflammation; elevated in TRD and associated with poor antidepressant response.	Predictor of response to anti-inflammatory therapies.
-	Serum Amyloid A (SAA)	Reflects chronic systemic inflammation contributing to neurodegeneration.	Emerging biomarker for inflammation-driven TRD.
Neuroimmune Enzymes	Indoleamine 2,3-Dioxygenase (IDO)	Promotes tryptophan metabolism into neurotoxic kynurenine metabolites, reducing serotonin availability.	Potential target for IDO inhibitors to restore serotonin balance.
-	Cyclooxygenase-2 (COX-2)	Increases prostaglandin production, amplifying neuroinflammation.	Targeted by COX-2 inhibitors (*e.g*., celecoxib).
Oxidative Stress Markers	Malondialdehyde (MDA)	Indicates lipid peroxidation due to oxidative stress, often elevated in TRD.	Used in research as a marker for oxidative neuroinflammation.
-	8-Hydroxy-2'-deoxyguanosine (8-OHdG)	Reflects DNA oxidative damage caused by chronic inflammation.	Marker for long-term neuroinflammatory damage.
Glial Activation	Translocator Protein (TSPO)	Elevated TSPO expression indicates microglial activation and neuroinflammation.	Measured using PET imaging, TSPO ligands are research tools for tracking inflammation in TRD.
Immune Cells	Neutrophil-to-Lymphocyte Ratio (NLR)	Elevated NLR correlates with systemic inflammation and depression severity.	Emerging peripheral marker for immune dysregulation in TRD.
Metabolites	Kynurenine Pathway Metabolites	High kynurenine-to-tryptophan ratios indicate inflammation-driven depletion of serotonin.	Biomarker for inflammation-driven TRD; potential therapeutic target.
-	Quinolinic Acid	Neurotoxic metabolite promoting NMDA receptor hyperactivation and neurodegeneration.	Marker for excitotoxicity and neuroinflammation in TRD.
Hormonal Indicators	Cortisol	Chronic elevation reflects HPA axis overactivation, which is linked to neuroinflammation.	Potential marker for stress-related neuroinflammation in TRD.
-	Dehydroepiandrosterone (DHEA)	Imbalances in cortisol-to-DHEA ratios indicate chronic inflammation and dysregulation of the HPA axis.	Used in conjunction with cortisol to assess inflammatory dysregulation.

**Table 2:** SWOT analysis of miRNA-based therapeutic strategies in treatment-resistant depression (TRD) this analysis outlines the diagnostic, therapeutic, and research potential of miRNAs in TRD treatment, along with the challenges, clinical limitations, and external factors influencing the development and application of miRNA-based therapies in precision psychiatry.

Strengths	Weaknesses
Diagnostic Potential: miRNAs offer reliable biomarkers for diagnosing TRD and predicting treatment responses. This is crucial for early intervention and personalized treatment approaches in TRD and other treatment-resistant psychiatric conditions.	Delivery Challenges: Effective miRNA delivery across the blood-brain barrier remains a significant challenge. Current delivery systems, such as nanoparticles or viral vectors, require further development to ensure safety and efficacy in the brain.
Therapeutic Modulation: miRNA mimics and antagomiRs provide versatile therapeutic tools that can be tailored to individual molecular profiles. This enables the targeting of specific pathways, such as serotonin signaling, inflammation, and neuroplasticity, to enhance treatment responses.	Long-Term Safety Concerns: The long-term effects of miRNA-based therapies on gene regulation and neurophysiology remain unclear, raising concerns about potential adverse effects resulting from prolonged modulation.
Personalization and Precision: miRNA profiling enables treatments to be tailored to an individual's genetic, epigenetic, and environmental factors, aligning with the shift toward precision psychiatry and enhancing patient outcomes.	Off-Target Effects: miRNAs often interact with multiple mRNA targets, which can lead to unintended genetic modulation, necessitating careful design of specificity to avoid unwanted changes in gene expression.
Research on Combination Therapies: The potential for miRNA-based interventions to complement established treatments, like SSRIs, ECT, and psychotherapy, presents opportunities to improve outcomes through combination therapies and synergistic effects.	Limited Clinical Data: While preclinical studies are promising, miRNA-based treatments for TRD lack extensive clinical validation. Further research is needed to confirm efficacy, determine optimal dosing, and assess safety in diverse patient populations.
**Opportunities**	**Threats**
Expansion to Other Psychiatric Disorders: miRNA-based treatments have applications beyond TRD, with potential impact on other psychiatric conditions, such as anxiety and bipolar disorder, where treatment resistance is common.	Regulatory and Ethical Hurdles: The novel nature of miRNA therapies poses regulatory challenges. Ethical considerations, such as potential long-term cognitive effects, may delay regulatory approvals and patient acceptance.
Advances in Delivery Systems: New technologies, such as engineered nanoparticles and exosome-based delivery, offer promising solutions to improve miRNA transport across the blood-brain barrier, increasing clinical applicability.	Immune Response Risks: Immune-mediated adverse effects, as seen in some miRNA clinical trials, raise concerns about patient safety and acceptance. These reactions require careful study and the development of mitigation strategies.
Role in Precision Psychiatry: Integrating miRNA biomarkers could help clinicians develop targeted treatment plans, fostering more effective interventions tailored to each patient's unique biological profile.	High Development Costs: The complexity of miRNA-based therapies necessitates substantial investment in research and development, which may hinder their widespread adoption and delay clinical availability.
Potential for Novel Therapeutics: Research into miRNAs may lead to the development of entirely new therapeutic approaches targeting other RNA molecules or gene pathways, broadening the scope of psychiatric treatment.	Variability in Patient Response: Genetic, environmental, and lifestyle factors that affect miRNA expression may lead to inconsistent responses to miRNA-based therapies, complicating the standardization of treatment.

**Table 3:** Overview of miRNAs implicated in treatment-resistant depression (TRD), target genes and proteins, and their associated functions. The table highlights miRNAs regulating critical pathways such as neuroplasticity (e.g., miR-1202, miR-124), inflammation (e.g., miR-146a), and apoptosis (e.g., miR-16, miR-21).

**miRNA**	**Target Genes**	**Proteins**	**Function/Role in TRD**
hsa-miR-1202	BDNF, TRKB	Brain-Derived Neurotrophic Factor (BDNF), Tropomyosin receptor kinase B (TRKB)	Implicated in neuroplasticity and mood regulation.
hsa-miR-135	5-HTT, GSK3B	Serotonin Transporter (5-HTT), Glycogen Synthase Kinase 3 Beta (GSK3B)	Modulates serotonin levels, influencing mood and depression.
hsa-miR-124	REST, BDNF	RE1-Silencing Transcription Factor (REST), BDNF	Regulates neuronal development and synaptic plasticity.
hsa-miR-16	BCL2, MCL1	B-cell Lymphoma 2 (BCL2), Myeloid Cell Leukemia 1 (MCL1)	Involved in apoptosis regulation; dysregulation linked to depression.
hsa-miR-146a	IL6, TNFα	Interleukin 6 (IL6), Tumor Necrosis Factor Alpha (TNFα)	Modulates inflammation pathways associated with depression.
hsa-miR-548ba	LIFR, PTEN, NEO1	Leukemia Inhibitory Factor Receptor (LIFR), Phosphatase and Tensin Homolog (PTEN)	Targets genes involved in neuroprotection and cell survival.
hsa-miR-7973	TGFBR2, ADAM19	Transforming Growth Factor Beta Receptor 2 (TGFBR2), ADAM Metallopeptidase Domain 19	Influences cellular signaling pathways related to stress responses.
let-7 Family	Dicer, TARBP2	Dicer, TAR RNA Binding Protein 2	Regulates miRNA biogenesis; altered expression linked to TRD.
hsa-miR-132	RASGRF1, P250GAP	RAS Protein Specific Guanine Nucleotide Exchange Factor 1 (RASGRF1), P250GAP	Involved in synaptic function and plasticity; linked to mood disorders.
hsa-miR-30a	SIRT1	Sirtuin 1	Regulates cellular stress response and apoptosis; implicated in mood regulation.
hsa-miR-21	PDCD4, PTEN	Programmed Cell Death 4 (PDCD4), PTEN	Involved in apoptosis and cell survival; dysregulation may contribute to TRD.
hsa-miR-29b	COL1A1, MMP2	Collagen Type I Alpha 1 Chain (COL1A1), Matrix Metalloproteinase 2 (MMP2)	Associated with extracellular matrix remodeling; potential role in neuroplasticity.

**Table 4:** Key miRNAs implicated in treatment-resistant depression (TRD) with their biological mechanisms and clinical relevance.

miR-135a	Regulates serotonin signaling pathways by targeting serotonin receptors and transporters	Modulation of miR-135a could improve responses to SSRIs by enhancing serotonin availability.	[65, 66]
miR-146a	Influences neuroinflammation by targeting components of the NF-κB signaling pathway	Potential biomarker for assessing inflammation levels in TRD patients; miR-146a modulation could reduce neuroinflammatory responses.	[45, 46, 67]
miR-34a	Associated with synaptic plasticity and neurogenesis	Upregulated in TRD and linked to cognitive decline; potential therapeutic target for enhancing neuroplasticity.	[68]
miR-124	Regulates neurogenesis and neuroplasticity pathways	Downregulated in depressive states; restoring miR-124 levels may enhance neuroplasticity and stress resilience.	[69]
miR-155	Modulates inflammatory responses by influencing immune pathways	Elevated in TRD; targeting miR-155 could reduce inflammation and improve treatment response.	[70]
miR-335-5p	Influences postsynaptic density and axonogenesis, which are key to neuronal communication	Significantly upregulated in TRD patients; may serve as a biomarker for diagnosis and monitoring.	[71]

**Table 5:** Diagnostic and predictive miRNAs for TRD, their roles, and clinical relevance.

miR-1202	Lower levels linked with non-response to SSRIs	Associated with reduced antidepressant efficacy, particularly in SSRIs	May serve as a biomarker for predicting SSRI treatment response in TRD patients	[122]
miR-146a	Elevated levels linked to inflammation in TRD	Could indicate the likelihood of non-response to standard antidepressants	Potential for monitoring inflammation and predicting treatment-resistant cases	[87]
miR-124	Downregulation is linked with neuroplasticity deficits	Indicates potential for better responses with neuroplasticity-focused therapies	Could help in selecting patients for therapies targeting neuroplasticity pathways	[41]
miR-155	Associated with inflammatory responses in TRD patients	Could identify patients with inflammation-driven TRD	Useful for personalized treatment approaches targeting inflammation in TRD	[51]
miR-135a	Involved in serotonin signaling; low levels linked with resistance	Predictive of poor SSRI response	May be used to guide SSRI-based treatment decisions in TRD patients	[66]

**Table 6:** Summary of key findings from longitudinal studies on miRNA expression in TRD.

miRNAs Studied	Findings	Clinical Implications	Duration	References
miR-135a, miR-155	Sustained upregulation of miR-135a correlated with improved SSRI response; increased miR-155 levels linked to inflammatory pathways and TRD cases.	Potential use of miR-135a as a positive prognostic marker and miR-155 as an indicator of inflammation-driven TRD.	18 months	[70, 116]
miR-146a, miR-34a	Fluctuations in miR-146a and miR-34a are associated with patient relapse; miR-34a remained elevated in TRD cases post-treatment.	miR-34a can serve as a stable marker for TRD relapse; miR-146a levels may aid in monitoring inflammation-based treatment responses.	12 months	[73, 116]
miR-1202, miR-124, let-7 family	Persistent downregulation of miR-1202 in TRD patients; let-7 family members decreased over time in non-responders to antidepressants.	miR-1202 as a predictor for SSRI non-response; let-7 downregulation may indicate ongoing neuroplastic deficits in TRD patients.	24 months	[127, 128]
miR-335-5p, miR-221	Increased miR-335-5p in non-responders; miR-221 expression linked to mood stabilization in responders over follow-up.	miR-335-5p as a non-response marker, while miR-221 could indicate a positive treatment response in TRD patients.	6 months	[51, 54]
miR-124, miR-132	miR-124 exhibited dynamic changes that aligned with cognitive improvement, while miR-132 correlated with long-term mood stabilization in TRD patients.	miR-124 as a marker for cognitive function restoration; miR-132 for tracking mood stabilization over extended periods.	36 months	[129-131]
miR-135a, miR-146a, miR-21	Consistent miR-135a increase in SSRI responders; miR-146a and miR-21 fluctuated with inflammatory episodes in TRD cases.	miR-135a is a favorable prognostic marker; miR-146a and miR-21 are useful for monitoring inflammation-related exacerbations and treatment adjustments.	9 months	[122, 132]
miR-155, miR-221, miR-34a	miR-155 linked to chronic inflammation; miR-221 downregulated post-ECT in responders; miR-34a elevated during remission phases in TRD patients.	Highlights the potential of miR-155 and miR-221 for tracking inflammatory and neuroplastic responses in TRD treatment.	15 months	[132, 133]

## LIST OF ABBREVIATIONS

AMPK: AMP-Activated Protein Kinase
BBB: Blood–Brain Barrier
BDNF: Brain-Derived Neurotrophic Factor
CNS: Central Nervous System
CSF: Cerebrospinal Fluid
EVs: Extracellular Vesicles
FDA: Food and Drug Administration
GABA: Gamma-Aminobutyric Acid
GWAS: Genome-Wide Association Studies
HPA: Hypothalamic–Pituitary–Adrenal
IL: Interleukin
IRAK: Interleukin-1 Receptor-Associated Kinase
MAOIs: Monoamine Oxidase Inhibitors
MAPK: Mitogen-Activated Protein Kinase
MDD: Major Depressive Disorder
miRNA: microRNA
mTOR: Mammalian Target of Rapamycin
NF-κB: Nuclear Factor Kappa-light-chain-enhancer of Activated B Cells
NNT: Number Needed to Treat
NPH: Normal Pressure Hydrocephalus
PET: Positron Emission Tomography
rTMS: Repetitive Transcranial Magnetic Stimulation
SERT: Serotonin Transporter
SIRT: Sirtuin
SNP: Single Nucleotide Polymorphism
SSRI: Selective Serotonin Reuptake Inhibitor
STAR*D: Sequenced Treatment Alternatives to Relieve Depression
TGF-β: Transforming Growth Factor Beta
TLR: Toll-like Receptor
TRAF: TNF Receptor Associated Factor
TRD: Treatment-Resistant Depression
WHO: World Health Organization
